# A spectrum of preferential flow alters solute mobility in soils

**DOI:** 10.1038/s41598-022-08241-w

**Published:** 2022-03-11

**Authors:** Jesse Radolinski, Hanh Le, Sheldon S. Hilaire, Kang Xia, Durelle Scott, Ryan D. Stewart

**Affiliations:** 1 School of Plant and Environmental Sciences, Virginia Polytechnic Institute, 185 Ag Quad Lane, Blacksburg, VA 24061 USA; 2 Department of Ecology, University of Innsbruck, Sternwartestraße 15A-6020, Innsbruck, Austria; 3 Department of Biological Systems Engineering, Virginia Polytechnic Institute, 155 Ag Quad Lane, Blacksburg, VA 24061 USA

**Keywords:** Environmental sciences, Environmental monitoring, Hydrology

## Abstract

Preferential flow reduces water residence times and allows rapid transport of pollutants such as organic contaminants. Thus, preferential flow is considered to reduce the influence of soil matrix-solute interactions during solute transport. While this claim may be true when rainfall directly follows solute application, forcing rapid chemical and physical disequilibrium, it has been perpetuated as a general feature of solute transport—regardless of the magnitude preferential flow. A small number of studies have alternatively shown that preferential transport of strongly sorbing solutes is reduced when solutes have time to diffuse and equilibrate within the soil matrix. Here we expand this inference by allowing solute sorption equilibrium to occur and exploring how physiochemical properties affect solute transport across a vast range of preferential flow. We applied deuterium-labeled rainfall to field plots containing manure spiked with eight common antibiotics with a range of affinity for the soil after 7 days of equilibration with the soil matrix and quantified preferential flow and solute transport using 48 soil pore water samplers spread along a hillslope. Based on > 700 measurements, our data showed that solute transport to lysimeters was similar—regardless of antibiotic affinity for soil—when preferential flow represented less than 15% of the total water flow. When preferential flow exceeded 15%, however, concentrations were higher for compounds with relatively low affinity for soil. We provide evidence that (1) bypassing water flow can select for compounds that are more easily released from the soil matrix, and (2) this phenomenon becomes more evident as the magnitude of preferential flow increases. We argue that considering the natural spectrum preferential flow as an explanatory variable to gauge the influence of soil matrix-solute interactions may improve parsimonious transport models.

## Introduction

A growing and increasingly affluent human population is releasing ever greater loads of organic contaminants into Earth’s critical zone^1^. Many of these compounds are susceptible to rapid movement via preferential flow^2–4^, which can often lead to orders of magnitude greater solute leaching than predicted by equations specific to a homogenous soil matrix (e.g., the advection–dispersion equation)^5,6^. Preferential flow is ubiquitous in soils^7^, representing as little as 1%^8^ to more than 70% of total water movement^9–11^. The associated potential for rapid chemical transport threatens water quality in nearby aquifers and streams, making it important to understand the key physical and chemical factors controlling organic compound movement through unsaturated soils^12^.

Preferential flow occurs as a disequilibrium between water flowing through the low-permeability bulk soil (i.e., the soil matrix) and the highly conductive fraction of the total soil volume such as macropores with hydraulic conductivities > 0.01 cm h^−1^^13^. Under such conditions, flow rates can sharply increase without uniform increases in soil pore water pressures^13–15^ as water bypasses the lower permeability matrix (i.e., bypass flow). This phenomenon is observed over a broad range of water contents^16–18^. In dry soils, preferential flow may occur as partially water-repellant layers destabilize the wetting front forming fingered flow^17^ or as flow is concentrated through newly-formed cracks^19,20^. As soils approach saturation, near-positive pore water pressures can force water from the matrix into highly conductive macropores^21–24^, making total flow proportionally more preferential^25^.

Preferential flow drastically reduces the residence time of water in the critical zone^26^, thus limiting the opportunity for dissolved substances to sorb to soil particles^27,28^. Therefore, when preferential flow is minimal, more homogenous flow through the soil matrix dominates, favoring transport of compounds with low affinity to soil, such as those with a low sorption coefficient (*K*_*d*_)^29–31^. Conventional considerations of preferential flow maintain that the influence of solute-matrix affinity decreases as flow becomes proportionally more preferential^32–36^ such that the kinetics of rapid flow restrict sorption^37^ or enhance desorption^38^. However, studies to date have mostly compared known preferential transport of solutes to more homogenous flow—either modeled based on advection–dispersion processes^39,40^ or focused on specific conditions such as frozen soils^41^—but have never directly assessed how organic contaminants of varying chemical properties become mobilized along a spectrum of flow heterogeneity. Relevant studies have primarily focused on precipitation-driven transport directly following solute application^13,18,28,32,33,36,42–47^, which—by forcing scenarios of rapid chemical and physical disequilibrium—may have perpetuated the view of preferential transport as a non-selective process (i.e., low influence of solute-matrix affinity). A small number of studies^48–50^ have noted that strongly sorbing solutes may be less susceptible to preferential leaching^51^ when rainfall timing is more lagged compared to solute application, facilitating more diffusion and sorption equilibrium within the soil matrix. Nevertheless, the conditions necessary to dampen versus amplify the influence of compound physiochemical properties on solute transport are not well understood. The primary objectives of this study were to (1) quantify transport of eight veterinary antibiotics under different preferential flow conditions and (2) determine if preferential flow can eliminate the influence of solute-soil affinity on transport of these solutes. This analysis is necessary to provide a fundamental understanding of how preferential flow alters contaminant mobility and build process-based transport models needed to manage water quality and thwart water resource degradation.

Here we explore the influence of solute-matrix affinity across a range of preferential flow by applying simulated rainfall to field plots containing manure spiked with eight common veterinary antibiotics (listed by decreasing relative affinity to the soil matrix): erythromycin (ERY), tylosin (TYL), tetracycline (TC), pirlimycin (PLY), chlortetracycline (CTC), oxytetracycline (OCT), sulfadimethazine (SDM), and sulfamethazine (SMZ). Veterinary antibiotics were chosen for (1) their environmental ubiquity, as up to 11.5 million kg were purchased in 2019 for livestock use alone in the U.S^52,53^. and animal waste applied to soils can contain 40–90% of these compounds in unaltered (not metabolized) form^54^, and (2) for their wide range of affinity to soils^55^. Antibiotic-spiked manure was applied to field plots (200 × 150 cm) on the soil surface or injected to a depth of 10 cm (*n* = 3 plots per application method). After 7 days of rainfall suppression, we applied rainfall at 7 cm h^−1^ (a standard and recommended rate for rainfall simulations^56^) to these plots, as well as to an additional 3 plots without treated manure that served as experimental controls. Rainfall simulations were labeled with deuterium to facilitate preferential flow quantification. Simulations were conducted until 0.5 h of continuous runoff was observed^56^, and in total lasted for an average of 1.2 h (Table S1). Monitoring soil pore water isotope signatures and antibiotic concentrations in suction lysimeters across time (1 h before, 30 min into, and 1 h) and space (multiple locations and depths of 30 and 90 cm) allowed us to produce > 700 solute transport observations along a spectrum of preferential flow. See “Methods” and Supporting information for more experimental details.

We defined antibiotic movement in terms of change in concentration, Δ*C*, from samples collected 0.5 h into and 1 h after rainfall versus pre-event (background) values from the same lysimeter. We deemed Δ*C* to be zero whenever veterinary antibiotic concentrations decreased from background or were non-detectable. At the same time, we considered flow to be partitioned into two distinct hydrological domains assuming faster advection through preferential pathways (e.g., root channels and macropores) versus slower flow through the soil matrix via combined advection and dispersion mechanisms. Following the conceptual framework provided by Stumpp, et al.^57^, the fractional contribution of preferential flow was calculated by ${f}_{PF}\left(t\right) =\frac{{D}_{t}\left(t\right)- {D}_{MF}\left(t\right)}{{D}_{PF}\left(t\right)- {D}_{MF}\left(t\right)}$, where sampled deuterium concentrations, *D*_*t*_(t), were used in a two-member mixing model that separated rainfall moving through preferential flow paths, *D*_*PF*_(t), from pre-event or mid-event soil matrix water, *D*_*MF*_(t) (see Methods for full derivation). We note that the average of 7 cm of rainfall infiltrated in this experiment (Table S1) would have replaced ~ 20 cm of storage via pure advection. This calculation suggests that a homogenous wetting front would not have reached our most shallow pore-water samplers (30 cm) and that the sampled water was derived from some combination of rainwater bypassing the soil matrix and pre-event matrix storage. As a result, we consider the mixing model to be suitable for quantifying preferential flow during the simulated rainfall experiment.

### Lysimeter measurements produce a spectrum of preferential flow

A total of 153 of the 768 (48 lysimeters × 2 effective measurements × 8 antibiotics) measurements (20%) resulted in zero or negative *f*_*PF*_ values, which we considered to represent entirely matrix-derived water (*f*_*PF*_ = 0) in subsequent analyses. Though event water was applied at a constant rainfall intensity (7 cm h^−1^) and infiltrated in similar rates between plots (Table S1), simulated rainfall produced nearly three orders of magnitude of variation in preferential flow (*f*_*PF*_ from 0.002 to 0.6; Fig. S1). The range of positive Δ*C* values extended nearly four orders of magnitude, from 0.006 to 3.9 µg L^−1^ (Fig. S2), with probability of detection highest in the low range of preferential flow (Fig. S3). These numerous point estimates of preferential flow in space (i.e., different lysimeter depths and random positions) and time (i.e., during and after rainfall) enabled analysis of solute mobility under a spectrum of flow heterogeneity.

**Figure 1:**
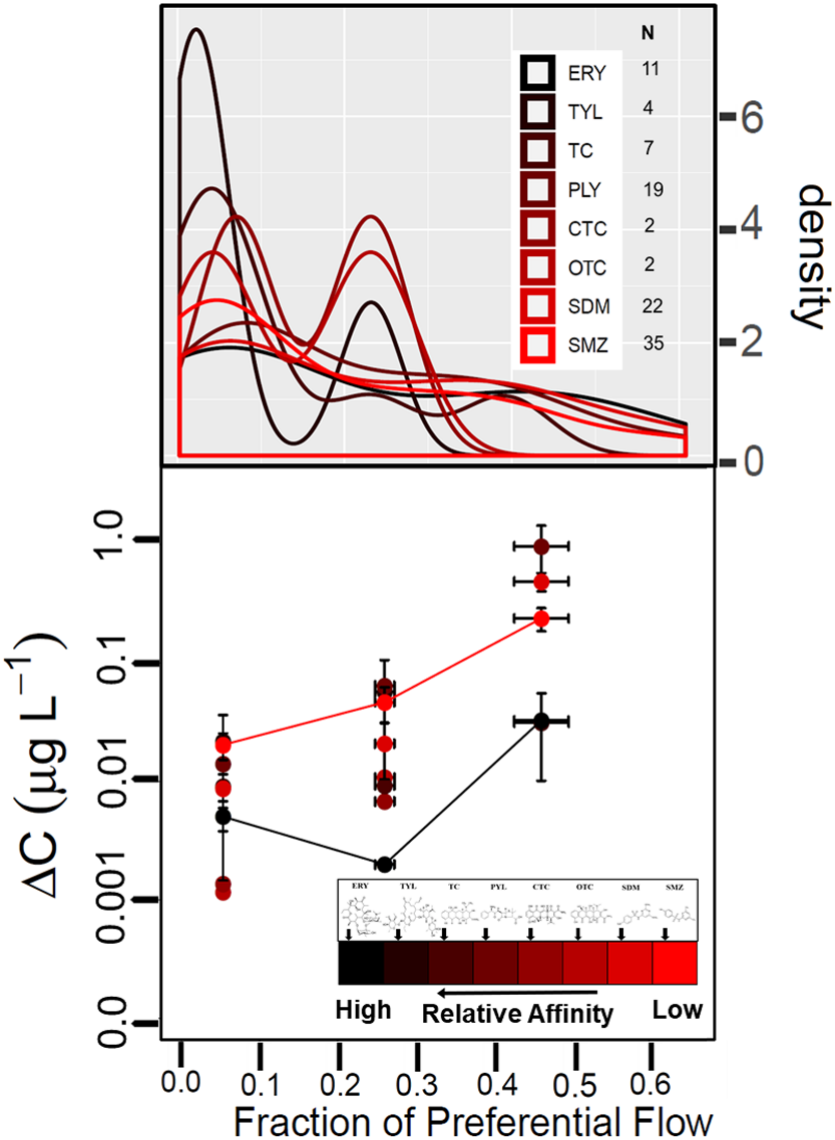
(**a**) Smoothed density distributions of tested antibiotics in the detected range of estimated preferential flow (x-axis), indicating the total frequency of samples detected; N = the total number of samples with detectable changes in antibiotic concentrations (Δ*C* > 0) and detectable preferential flow. (**b**) Change in antibiotic concentration (Δ*C*) when *f*_*PF*_ > 0 as binned using three ranges of preferential flow with highest densities (*f*_*PF*_ = 0–0.2, 0.2–0.35, and 0.35–0.61). Error bars represent standard error of the mean (SE). Colors indicate relative affinity to soil as ranked based on the sorption study (*K*_*d*_ values listed in Table S2): red indicates the compound with the lowest affinity (SMZ) and black indicates the compound with highest affinity (ERY) to soil. Lines track SMZ and ERY. R v3.5.2 was used to plot this figure^61^.

A frequency analysis of samples with detectable changes in antibiotic concentration (Δ*C* > 0) showed clustering in three distinct ranges of preferential flow: 0 < *f*_*PF*_ ≤ 0.15, 0.15 < *f*_*PF*_ ≤ 0.35, and 0.35 < *f*_*PF*_ ≤ 0.61 (Fig. 1a and Fig. S4; also see Fig. S2). In general, solutes with high relative affinity for the soil matrix, such as ERY, TYL, and TC, were most frequently detected under low preferential flow conditions (i.e., *f*_*PF*_ ≤ 0.15). The relatively low-affinity sulfonamides (SDM and SMZ) had a more uniform distribution across the range of preferential flow. While the high-affinity macrolides (ERY and PLY) had similar distributions as the sulfonamides, it should be noted that they were detected less often (N = 11 for ERY and 19 for PLY versus N = 22 for SDM and 35 for SMZ). Further, the sulfonamides were always detected under high preferential flow conditions (e.g., *f*_*PF*_ > 0.4), whereas ERY and PLY continued to have non-detects (i.e., Δ*C* = 0) in that range (Fig. S5). Altogether, these results suggest that bypass flow preferentially mobilizes some solutes over others, with relative affinity to the matrix acting as an important factor in this process.

### Influence of relative affinity is asymmetric across measured range from preferential flow

After binning data into the three preferential flow ranges (0 < *f*_*PF*_ ≤ 0.15, 0.15 < *f*_*PF*_ ≤ 0.35, and 0.35 < *f*_*PF*_ ≤ 0.61), the compounds with the greatest contrast in relative affinity to soil (e.g., the high-affinity macrolides TYL and ERY versus the low-affinity sulfonamides SDM and SMZ) were similar in Δ*C* for the low range of preferential flow, but diverged with increasing bypass flow (Fig. 1b). For example, when *f*_*PF*_ was < 0.15, TYL and SMZ had nearly identical Δ*C* values. If we were to simply lump Δ*C* values by compound class for the lowest (SMZ and SDM) and highest (ERY and TYL) relative affinity using the data in Fig. 1b, the two classes would be also similar in average Δ*C*: 0.014 µg L^−1^ for sulfonamides versus 0.013 µg L^−1^ for macrolides when *f*_*PF*_ was < 0.15. However, when preferential flow exceeded 0.35, Δ*C* was more than an order of magnitude higher for SDM compared to ERY. This finding suggests that the influence of solute-matrix affinity on transport was weakest when bypass flow was minimal.

Here we note that the antibiotic PLY, which had a relatively moderate affinity for soil, produced the highest Δ*C* in drainage in the high range of *f*_*PF*_ (Figs. 1b). However, a previous antibiotic transport study conducted in the same field site reported PLY as being highly mobile with 50 × more PLY transported in runoff compared to the sulfonamide SMZ^58^. This result suggests that PLY sorption to the A_p_ soil sample used for *K*_*d*_ determination may not have been representative of the entire field, or else that our ranking was accurate and the high apparent mobility of PLY seen in field-runoff studies^58,59^ reflects the influence of other controls on transport (such as colloidal transport, as discussed in the Supporting Information). Some numerical simulations^32,60^ and one recent column study^41^ have also suggested that solutes with moderate affinity for soil may be most susceptible to preferential flow. Though the underlying mechanisms are not yet clear, we speculate that these compounds may have high enough *K*_*d*_ to be sorbed throughout the soil medium, yet soluble enough to be partitioned or displaced into local bypass flow. Thus, when *f*_*PF*_ approached ~ 0.5 (i.e., roughly equal matrix and preferential contributions to flow), compounds with moderate relative affinity for soil could be selected in higher proportions relative to other antibiotics.

**Figure 2:**
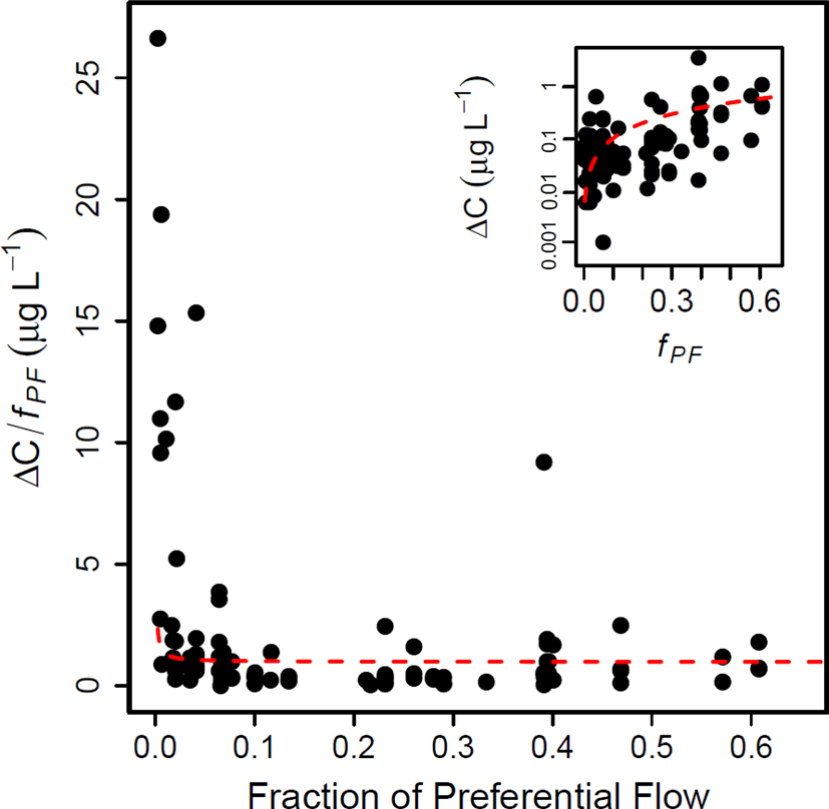
Solute susceptibility to preferential flow (Δ*C* /*f*_*PF*_) across the detected range of preferential flow. The red dashed line depicts a linear fit to raw (not log-transformed) data using all antibiotics (Δ*C* = 0 and *f*_*PF*_ = 0 excluded). The linear fits indicate a possible condition where antibiotics have constant susceptibility to leaching regardless of the amount preferential flow. Note that the y-axis in the inset figure has a logarithmic scale. R v3.5.2 was used to plot this figure^61^.

Solute transport to our lysimeters also appears to have been most susceptible to preferential flow (i.e., Δ*C*/*f*_*PF*_ was highest) when *f*_*PF*_ was < 0.15 (Fig. 2). In this low *f*_*PF*_ range, a low magnitude of preferential flow resulted in disproportionately high Δ*C* values. As drainage approached medium (0.15 < *f*_*PF*_ ≤ 0.35) and high (0.35 < *f*_*PF*_ ≤ 0.61) *f*_*PF*_ values, Δ*C* susceptibility to preferential flow was relatively constant. The influence of preferential flow on the magnitude of Δ*C* was therefore dampened when solute-matrix affinity became more influential, indicating a fundamental shift in solute and flow partitioning. For example, in a situation where Δ*C* linearly increased across the range of *f*_*PF*_ values (red dashed line fit to raw data in Fig. 2), the antibiotic detection in drainage would respond similarly (i.e., nearly constant Δ*C*/*f*_*PF*_) across the spectrum of preferential flow. Instead, Δ*C*/*f*_*PF*_ remained relatively constant above preferential flow values of 0.15 and Δ*C* was an order of magnitude more susceptible to preferential flow at the lowest versus highest *f*_*PF*_ values. These data thus further illustrate that solute responses to bypass flow differ along the spectrum of preferential flow, and more specifically, that a shift from non-selective to selective transport coincided with a decrease in overall Δ*C* susceptibility to preferential flow at the length scale of the lysimeters.

### Preferential flow triggers selective and non-selective transport

These differences in transport behavior can be explained by both the amount of preferential flow and the ability of this bypass flow to access antibiotics. For example, 7 days of rainfall suppression would likely have been sufficient time for compounds to diffuse into the soil matrix^13,48,62–64^ and for sorption equilibrium to occur^58,65^. Consequently, solute transport in plots spiked with antibiotics was nearly identical to control plots (Fig. S6), suggesting that these compounds may be stored in the soil matrix from previous applications. Therefore, when *f*_*PF*_ < 0.15, the likelihood of sampling all compounds was higher (Fig. 1a and Fig. S3) as most of the drainage water originated in the matrix. Infiltrating water may have mixed with a greater volume of pre-event storage before triggering preferential flow events with trace levels of antibiotics, allowing for compounds strongly sorbed to the soil matrix (e.g., high relative affinity) and compounds weakly bound to macropore walls (e.g., low relative affinity) to be transported in similar proportions. In contrast, higher proportions of preferential flow would have excluded flow through the matrix where much of the compounds resided^48,50,51,66,67^, causing the fast preferential flow domain to become more distinct from the slow matrix flow domain^68–70^ and infiltrating water to select for compounds with a higher affinity for the aqueous phase.

We additionally note that initiation of macropore flow often requires contributions from the soil matrix^21^, with the potential to dilute or displace the tracer signal in preferential flow paths^23,71^. This process can lead to underestimations of event water contributions to preferential flow^23,24,71^. Our method may not have distinguished these preferential flow scenarios from matrix water; rather, our analyses were intentionally focused on preferential flow paths that originated at or near the soil surface. Under the assumption that antibiotics were near the surface at the time of rainfall (max manure injection depth of 10 cm), our *f*_*PF*_ calculations would have detected fast-flowing event water contributions with the greatest potential to rapidly transport these solutes to depth. High Δ*C* at large values of *f*_*PF*_ (e.g., Fig. S6) also suggest that tracer dilution via displacement mechanisms may have been limited. Altogether, our *f*_*PF*_ estimates should provide a useful representation of flow heterogeneity and identify source contribution of water and solutes in drainage. Further, we encourage the use of alternative preferential flow detection methods^5^, under similar experimental conditions, to determine the relevance of this range of detected preferential flow—and its bearing on relative solute transport—in other heterogeneous systems.

### A revised understanding: treating preferential flow as an explanatory variable

**Figure 3:**
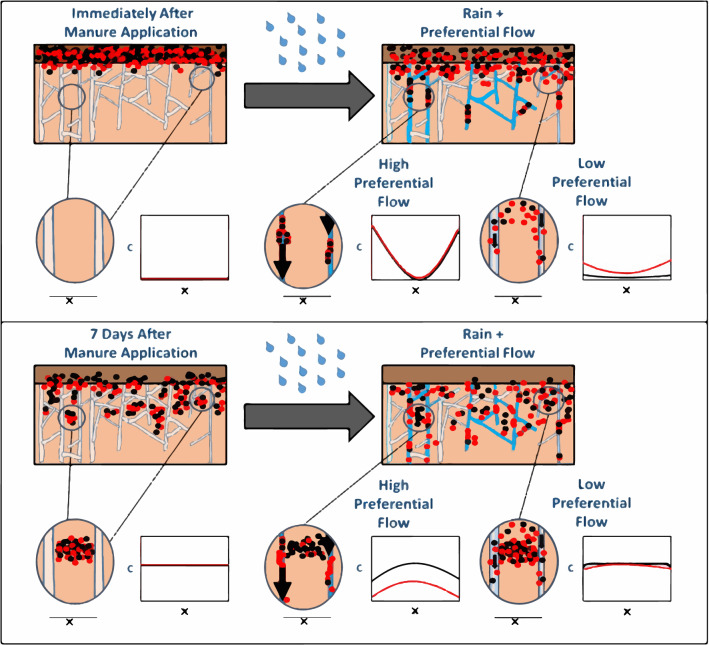
Different subsurface partitioning scenarios of solutes (dots) with high (black) and low (red) relative affinity to soil. Hypothetical solute concentration profiles (C vs x) are expressed at arbitrary locations spanning macropores surrounding a portion of the soil matrix. The top panel illustrates how both solutes would behave if rainfall simulations were conducted on the same day as antibiotic-spiked manure was applied. Compounds would have limited time to infiltrate into the soil matrix and come into sorption equilibrium, and high amounts of bypass flow through macropores could sample both compounds regardless of their relative affinity for soil. The bottom panel describes our experimental results, in which simulated rainfall occurred on the 7th day after antibiotic-spiked manure was applied to the plots. In this scenario, the elapsed time allowed solutes to diffuse into the soil matrix and sorption equilibrium to occur, so drainage with greatest macropore contributions (high preferential flow) could select for compounds with low-affinity for the soil. As a result, more residue would be found in the soil matrix for high versus low affinity compounds where high preferential flow occurred. In contrast, drainage with higher matrix contributions (low preferential flow) could sample all compounds in similar proportions, since the matrix concentrations were likely more similar between high and low affinity solutes.

In this study we treated preferential flow as an explanatory variable, which revealed that conventional transport phenomena may hinge on the degree of flow heterogeneity. This distinction appears to be unprecedented in the literature, in part, because none have considered how the magnitude of preferential flow alters the influence of solute-matrix affinity in soils. As a result, these findings contradict previous depictions of solute transport, where the influence of compound properties was thought to be significantly reduced with bypass flow^13,32,33,36,42–45,72,73^. To further explore this result, we used the conventional dual permeability model framework of Gerke and Van Genuchten^74^ with the HYDRUS 1D^75^ numerical platform to simulate analogous conditions to our experimental design (See Supplemental Information for details). Modeling results clearly predict that the difference in transport between solutes of high and low relative affinity decreased as the fraction of preferential flow increased (See Fig. S7, and Tables S2, S3, and S4). In contrast, our data indicated that when preferential flow intensified, Δ*C* in drainage became more influenced by the physiochemical interactions with the medium rather than just the medium itself (Fig. 3).

### Despite some uncertainties, study results are transferable

Our approach included some sources of uncertainty. First, the study included only one simulated storm with a constant rainfall intensity, and only considered one interval between manure application and simulated rainfall (7 days). This approach meant we missed the opportunity to study precipitation-driven transport shortly after manure application, where the influence of compound properties would likely have been enhanced^50,51^. Using a similar experimental design, Le, et al.^58^ detected comparable losses to runoff for four antibiotics of varying mobility when rainfall occurred just 2 h after manure application, yet losses differed by an order of magnitude when manure was undisturbed for 3 days. Therefore, the timing of precipitation appears to be an important factor controlling compound behavior in the presence of preferential flow, due to sorption kinetics and physical partitioning of the compounds below-ground. Since organic contaminants in soil often reach sorption equilibrium within a few days^76,77^, farmers can take advantage of this selective transport phenomenon by applying manure during periods without forecasted rain.

Another potential source of uncertainty was that relatively few of our samples came from high preferential flow conditions (Fig. S3). This limitation meant that observations collected under high preferential flow had disproportionately greater weighting on the overall trend than those under low preferential flow (e.g., Fig. S6). At the same time, the background detection of five antibiotics (e.g., Fig. S9) added some uncertainty to the results within low Δ*C* range. Nonetheless, we did not observe any preference for compounds based on their relative affinity in this range, suggesting that these sources of uncertainty did not strongly influence our interpretation. We also calculated Δ*C* (and *f*_*PF*_) on a per-lysimeter basis rather than using a difference from a mean background level, thus avoiding uncertainty that would arise from a lumped field-scale metric (see “Methods” for further details). Though negative *f*_*PF*_ values do add some uncertainty to preferential flow estimation, these values were relatively low (0.04 on average). This not only supports our assumption of *f*_*PF*_ = 0 for these samples but also suggests that susceptibility Δ*C* (Fig. 2) in the low range of preferential flow is unlikely to be the result of mixing model errors. It is also likely that our suction lysimeters did not intercept all heterogenous flow paths within the near-surface soil, particularly since preferential flow often acts as a stochastic phenomenon^78^ that stems from activation of a small percentage of the total soil porosity^11,79^. Even so, the wide range of antibiotic concentrations and preferential flow proportions detected in the water samples imply that these observations adequately represented non-equilibrium antibiotic transport at the site.

Despite the aforementioned sources of uncertainty, the study was able to encompass a range of experimental conditions and outcomes, including that 1) the storm produced preferential flow estimates spanning 3 orders of magnitude, 2) the relative affinity of our eight antibiotics differed by up to two orders of magnitude, and 3) our analysis included > 700 measurements of antibiotic concentrations and preferential flow proportion. As a result, the conceptual framework developed in this study (Fig. 3) should translate to other macroporous soils and transport scenarios.

### Implications

It has long been known that preferential flow can non-selectively transport a range of compounds^13,33,39,40,80^. Moreover, solute diffusion and sorption equilibrium within the soil matrix may limit subsequent transport through preferential flow paths when rainfall timing is lagged relative to chemical application^48,50,64,67^, with strongly sorbing substances often being the most affected^51^. However, the specific conditions necessary to dampen versus amplify the influence of compound physiochemical properties on solute transport remain poorly understood. By stimulating a range of preferential flow conditions in this study, we were able to quantify constraints on subsurface solute movement and partitioning. Our results indicate that preferential flow reduces the influence of soil matrix-solute interactions in soils^13,32,33,36,42–46^; yet we show that this influence can be distorted asymmetrically across the detectable range of preferential flow.

Our results suggest that under field-relevant scenarios the influence of solute-chemical properties were damped below ~ 15% preferential flow, but amplified at higher contributions of event water. Mechanistically, this contrast means that fast flow paths may preferentially select for solutes with low matrix affinity. Practically, this finding implies that soil and solute physicochemical properties may become more, not less, influential as the magnitude of preferential flow increases. One implication of this result is that traditional reactive transport models (e.g., single domain flow and single sorption site) may sufficiently describe solute movement under conditions of high, rather than minimal, preferential flow. At the same time, explicitly modifying the influence soil matrix-solute interactions along a spectrum of preferential flow may improve parsimonious transport models. Given the ubiquity of preferential flow observations^8,9,18,81,82^, these findings are necessary to develop better strategies for retaining mobile contaminants within the soil profile.

## Methods

### Field study site and preparation

The field experiment was conducted in the spring of 2018 on a no-till agricultural field in Whitethorne, Virginia. The field had a 9 to 11% slope and was underlain by two loam-textured soil series: Braddock and Unison (Typic Hapludults) with moderate soil structure. Soil physiochemical and hydraulic properties methods are described in Table S3. A total of nine randomly placed rainfall simulation plots were installed in the field. Each plot consisted of a 200 × 150 cm steel frame inserted 10 cm into the soil surface, with adjacent 40 cm × 200 cm buffer strips maintained outside of the frame for installation of soil pore-water samplers. A steel pan was fitted to each frame, sealed for runoff collection, and piped down-gradient to a container for storage and quantification (Fig. S8). Weed growth was then suppressed in all plots with glyphosate, to limit interception of rain and manure with vegetation and reduce evapotranspiration for the simulation period. Plots were differentiated into two treatments whereby manure was homogenously broadcasted on the soil surface (surface application; *n* = 3 plots) or injected below-ground into two 5 cm wide × 10 cm deep slits placed perpendicular to the slope and spanning the width of the plot frame and buffer strip (subsurface injection; *n* = 3 plots). The three remaining plots were used as controls by avoiding manure application and input of antibiotics. However, we detected some residual antibiotics in the control plots, which likely remained from manure applications in previous years (Fig. S9). The presence of residue antibiotics in the control plots provided us with the opportunity to assess the mobility of compounds under short (up to 7 days) and long term (e.g., greater than 6 months) equilibration with the soil matrix.

Prior to manure application, we installed a series of suction lysimeters (200 kPa ceramic cups; Soil Moisture Equipment Corp., Santa Barbara, CA) in the plot buffer strips to sample veterinary antibiotic transport in the subsurface. These buffer strips received the same amount of rainfall and manure treatment yet were located outside of the metal plot frames. Soil pore water samples were withdrawn from two randomly positioned lysimeters in both the Bt1 (30 cm) and Bt2 horizons (90 cm) to detect vertical movement of antibiotics in surface application plots, with two probes per depth making four probes per plot (Figs. S8 and S10). This same installation scheme was also adopted for control plots. In subsurface injection plots a series of nested (30 and 90 cm probes) lysimeters were installed both within and 25 cm down-gradient of the injection slit to detect vertical and lateral transport of antibiotics, with two probes per depth resulting in eight probes per plot.

A liquid slurry of dairy manure (5% solid content) was spiked with eight commonly used antibiotics: two macrolides, erythromycin and tylosin; two sulfonamides, sulfamethazine and sulfadimethoxine; three tetracyclines, oxytetracycline, chlortetracycline, and tetracycline; and one lincosamide, pirlimycin. A target concentration of 500 µg L^−1^ was used for all antibiotics. The spiked dairy manure slurry was then applied to each plot 7 days prior to rainfall simulations at a rate of 56 Mg wet mass ha^−1^^58^. If natural rainfall occurred within the 7-day equilibration period, the plots were covered with plastic tarps to prevent unintentional water input to the plots^58^.

### Assessment of antibiotic relative affinity to soil

We performed a simple solute partitioning test in the laboratory to determine the linear sorption coefficient, *K*_*d*_, for each of the eight antibiotics with soil from the field site (details in the Supplemental Information). Under this framework, each antibiotic was assigned a rank from 1 (highest affinity) to 8 (lowest affinity) based on measured *K*_*d*_ values (Table S5). The antibiotics ERY and TYL were not detectable in the supernatant during the test, so were given respective rankings of 1 and 2. Additionally, we used the USEPA’s BIOWIN model from the EPI (estimation program interface) Suite tool^83^ to estimate dissipation half-lives of each compound in soil following the methods described in Chen, et al.^84^. Using these half-lives we projected that less than 10% of the originally applied antibiotic mass would have degraded during our 7-day experiments, so we therefore assumed that decay played a minor role during transport.

### Field rainfall simulations and water sampling

After the 7-day equilibration period, we conducted rainfall simulations using deuterium-labeled well water to trace mobile infiltrating water and detect preferential flow contributions to pore water signature. The rainfall simulator (240 cm × 300 cm) followed the original design of Humphry, et al.^85^, which has been adopted as standard protocol for the national research project for simulated rainfall-surface runoff studies^56^ because it provides constant droplet size and velocity between locations and studies. We conducted the rainfall simulations with the SERA-17 standard intensity of 7 cm h^−1^, with rainfall continuing on each plot until the collection containers received 30 min of continuous runoff. Rainwater was isotopically labeled using a Dealglad venturi injector (9.0 × 5.5 × 5.5 cm; Shandong Jiujin Plastic Products Co., Shandong, China) fitted to the sprinkler inlet. This system dispensed an enriched deuterium solution into the well water at a desired ratio of ~ 4:100 (deuterium-spiked water: well water). Discrete pore water samples were taken from all lysimeters by applying 60 kPa of suction for 10 min, with samples collected 1 h before the simulation, 0.5 h into the simulation, and 1 h after the simulation (Fig. S10). All liquid samples were analyzed for ^2^H via cavity ring down spectroscopy (Model L1102-i, Picarro, Santa Clara, CA) and for all eight antibiotics via HPLC MS/MS, as detailed in the Supporting Information. To understand how these preferential flow estimates affected the transport of our eight veterinary antibiotics with a spectrum of relative affinity for soil, we quantified the change in concentration from lysimeter samples collected before versus during and after simulation (Δ*C*) as a function of *f*_*PF*_.

Here we note some potential constraints of using suction cup sampling to represent soil pore water. For example, suction lysimeters often have a smaller volume-of-influence compared to alternative pore water samplers^86–88^, reducing the likelihood of intercepting every preferential flow path below the plots. Suction cups can also have biased representation of water in larger—more “mobile”—pores^89–91^. We note that, though matrix and macropore waters can resist mixing during extreme rainfall^92^, complete mixing between pores can occur within days^92–94^. Thus, point measurements from our samplers (after 7 days of rainfall exclusion and equilibration) likely yielded representative samples of pre-event water from the matrix and labeled event water from mobile water in maropores, while capturing a wide range of stable isotope signatures.

### Preferential flow analysis

We considered flow to be partitioned into two distinct hydrological domains: faster advection through preferential pathways (e.g., root channels and macropores) and slower flow through the soil matrix via combined advection and dispersion mechanisms. Following the conceptual framework provided by Stumpp, et al.^57^ the isotope mass balance can be described as:

1 $${Q}_{t}\left(t\right)={Q}_{PF}(t)+ {Q}_{MF}(t)$$

And:

2 $${Q}_{t}\left(t\right)\cdot {D}_{t}\left(t\right) ={Q}_{PF}(t)\cdot {D}_{PF}\left(t\right)+ {Q}_{MF}(t)\cdot {D}_{MF}\left(t\right)$$

where the preferential flow, *Q*_*PF*_(*t*), and matrix flow, *Q*_*MF*_(*t*), equal total discharge *Q*_*t*_(*t*) [L^3^ T^−1^], and *D*_*MF*_(*t*)*, D*_*PF*_(*t*)*,* and *D*_*t*_(*t*) [M L^−3^] correspond to the isotope concentrations within each flow component. Here *D*_*PF*_(*t*) was set equal to pre-event and mid-event isotope lysimeter signatures for samples taken 0.5 into and 1 h after rainfall, respectively. Assuming that preferential flow pathways translate to rainfall inputs during each sampling period, we consider the rainfall isotope signal to be equivalent to the preferential flow signal in the outlet^57,95^:

3 $${D}_{PF}\left(t\right) ={D}_{rain}\left(t\right)$$

The total isotope concentration, *D*_*t*_(*t*)*,* detected in the outlet is equivalent to:

4 $${D}_{t}\left(t\right)= \frac{{Q}_{PF}(t)\cdot {D}_{PF}\left(t\right)+ {Q}_{MF}(t)\cdot {D}_{MF}\left(t\right)}{{Q}_{t}\left(t\right)}= {f}_{PF}(t)\cdot {D}_{PF}\left(t\right)+ {f}_{MF}(t)\cdot {D}_{MF}\left(t\right)$$

Since the fraction of matrix flow *f*_*MF*_(*t*) = 1 − *f*_*PF*_(*t*), Eq. (4) can be rearranged to describe the fractional contribution of preferential flow to the outlet signal as:

5 $${f}_{PF}\left(t\right) =\frac{{D}_{t}\left(t\right)- {D}_{MF}\left(t\right)}{{D}_{PF}\left(t\right)- {D}_{MF}\left(t\right)}$$

and the preferential flow rate as:

6 $${Q}_{PF}\left(t\right) ={f}_{PF}\left(t\right)\cdot {Q}_{t}\left(t\right)$$

We also note that mass transfer between the slow flowing matrix water is implicitly considered in the mixing model. For example, we can consider a scenario where event water infiltrates into the soil matrix and spills into a preferential flow path yielding an *f*_*PF*_ value of 0.50. Because event water reached the outlet before the wetting front it must have required preferential transport and thus 50% of the total water outflow is deemed preferential; with the remainder derived from pre-event matrix water.

### Reactive transport and experimental perspective

By applying labeled rainfall simulations to a heterogeneous no-till soil containing manure spiked with 8 antibiotics, we were able to quantify the amount of preferential flow in lysimeter drainage and assess the influence of compound properties on solute transport using the compounds’ wide range of relative affinity to the matrix. Additionally, because (1) the mass of antibiotics applied in manure was consistent between compounds and manure treatment (surface application versus subsurface injection), (2) estimated half-lives (described above) suggest that degradation was a minimal over the 7-day equilibration period, and (3) we were not concerned with metabolites of these antibiotics, our analysis did not require the explicit use of reactive transport models.

Our analysis was primarily focused on conditions where *f*_*PF*_ was positive, such that we could observe when and if preferentially flowing water contained relevant (> background levels) levels of antibiotics. This filtering distills large sample numbers into the most relevant values, removing unnecessary variability in solute transport from analysis. Our analysis in Fig. 1b includes all points where *f*_*PF*_ > 0. Thus, points where ΔC = 0 could bring down the observed average accordingly, giving a more representative depiction of how preferential flow contributed to the relative transport of antibiotics.

### Statistical analysis and data processing

We used a two-way analysis of covariance (ANCOVA) to statistically compare the slope of lines fitted to log-transformed Δ*C* as function of preferential flow (*f*_*PF*_ > 0 and Δ*C* > 0) by manure application treatment (i.e., subsurface injection, surface application, and control plots with only levels of antibiotics) and lysimeter depth (30 cm vs 90 cm). Specifically, this allowed us to identify the statistical significance of manure treatment on Δ*C* across the observed range of the covariate (preferential estimates) while also testing for the interaction of depth on this relationship. The log-transformed data were found to meet ANOVA assumptions of normality (via normal-quantile plots) and homogeneity of variances (via Fligner’s test). We used R version 3.5.2^61^ to conduct all statistical analyses with α = 0.05. We found no significant difference between the slope of lines fitted to Δ*C* data across the range of *f*_*PF*_ for all treatments, and no significant influence of lysimeter depth on this relationship (see Supplemental Information and Fig. S11). Additionally, because (1) high variation in *K*_s_ across our field (Table S6) suggested that intrinsic flow heterogeneity would mask any influence of time on Δ*C*, and 2) there appears to be no consistent influence of sampling time on the relationship between Δ*C* and *f*_*PF*_ (Fig. S12), we considered the effect of sampling time to be negligible on this relationship (though, we note that 0.5 h sampling restricted *f*_*PF*_ to below 0.30). Thus, we compiled all data together for subsequent analyses without separating treatment or depth.

## Supplementary Information

Supplementary Information 1.

Supplementary Information 2.
