# Supplementary material for: A spectrum of preferential flow alters solute mobility in soils
Source: Sci Rep. 2022 Mar 11;12:4261. doi: 10.1038/s41598-022-08241-w (PMC8917131; doi:10.1038/s41598-022-08241-w)
Supplement: Supplementary file 2 — Supplementary Information 2. [file 41598_2022_8241_MOESM2_ESM.docx]

**Supporting Information: A spectrum of preferential flow alters solute mobility in soils**

**This file contains 25 pages including 8 tables and 11 figures.**

Rainfall Simulation Details and Preferential Flow Discussion Page S2

Range of Preferential Flow Page S3

Distribution of Δ*C* versus *f_PF_*  Page S4

Distribution of Detectable (∆*C* > 0) and Non-Detectable (∆*C* = 0) Values Page S6

Soil Colloidal Transport and Other Controlling Factors Page S8

Assessment of Antibiotic Relative Affinity to Soil Page S9

Sulfonamides in Control Plots Page S10

Soil Sampling Page S10

Comparison with Conventional Dual Permeability Simulations Page S12

Analytical Approach Page S16

Field Plot Design and Lysimeter Placement Page S19

Background Detection of Antibiotics Page S21

Lysimeter Sampling Details Page S22

Manure Treatment Comparisons and Sampling Time Page S22 Water Samples Taken Page S24

References Page S26

**Rainfall Simulation Details and Preferential Flow Discussion**

Rainfall simulations lasted for an average of 1.2 h and generated 8 cm of cumulative rainfall and 7 cm of infiltration, with the remainder of water leaving plots as surface runoff (**Table S1**). Soil water content in the near-surface (0-5 cm) increased from 0.31 cm^3^ cm^-3^ prior to rainfall to 0.37 cm^3^ cm^-3^ immediately after rainfall. Under the assumption that event water infiltrated vertically via pure advection, the average of 7 cm of infiltrated water would have filled and displaced ~20 cm of storage. ^1,2^ This estimate suggests that a homogenous wetting front would not have reached our 30 cm lysimeters, and that the sampled water was derived from some combination of preferential flow and pre-event matrix storage. Thus, our assumption that event water seen in lysimeter samples represented preferential appears to be valid for this experiment. We also note that mass transfer between the slow flowing matrix water is implicitly considered in the mixing model. For example, we can consider a scenario where event water infiltrates into the soil matrix and spills into a preferential flow path yielding an *f_PF_* value of 0.50. Because event water reached the outlet before the wetting front it must have required preferential transport and thus 50% of the total water outflow is deemed preferential, with the remainder derived from pre-event matrix water. The δ^2^H difference between individual pore water samples taken before the simulation was relatively small (standard deviation of 5 ‰ from all plots and depths) compared to the difference between these samples and the labeled rainfall (average difference of 26 ‰; **Table S1**). Thus, we assumed that background lysimeter samples represented a uniform pre-event matrix signature *C_MF_*(t) which enabled calculation of *f_PF_* during and after field rainfall simulations for each lysimeter.

**Table S1.** Rainfall simulation information expressed as mean ± standard deviation. *θ* = soil volumetric water content from 0-5 cm soil depths.

| **Rainfall Simulations** | | |  |
| --- | --- | --- | --- |
| ***Time*** | Length of Simulation (h) | 1.2 ± 0.56 |  |
|  | Time to Runoff (h) | 0.75 ± 0.53 |  |
|  |  |  |  |
| ***Rates*** | Rainfall Rate (cm h^-1^) | 7 |  |
|  | Infiltration Rate (cm h^-1^) | 6 ± 0.8 |  |
|  |  |  |  |
| ***Depths*** | Rainfall (cm) | 8 ± 4 |  |
|  | Infiltration (cm) | 7 ± 3 |  |
|  | Runoff (cm) | 1.8 ± 1.3 |  |
|  |  |  |  |
| ***Tracer*** | Rainfall Label δ^2^H (‰) | -12.7 ± 8.44 |  |
|  | Background Pore Water δ^2^H (‰) | -39 ± 5.0 |  |
|  |  |  |  |
| ***θ*** | 1 h Before Simulation (cm^3^ cm^-3^) | 0.31 ± 0.043 |  |
|  | 1 h After Simulation (cm^3^ cm^-3^) | 0.37 ± 0.049 |  |

**Range of Preferential Flow**

Based on our detection method, negative values indicated that our lysimeter samples taken during and after the simulation were equal to or more isotopically depleted than the estimated matrix signature (i.e., samples pulled ~1 h before simulated rainfall). Negative *f_PF_* values suggest a dominant contribution of pre-event water, so we considered these points to be entirely matrix derived (*f_PF_* = 0). Including non-detect values caused a slight upward shift in the distribution of *f_PF_*, as seen by the Isotope (Positive) versus Isotope (All) data (**Figure S1**).

**
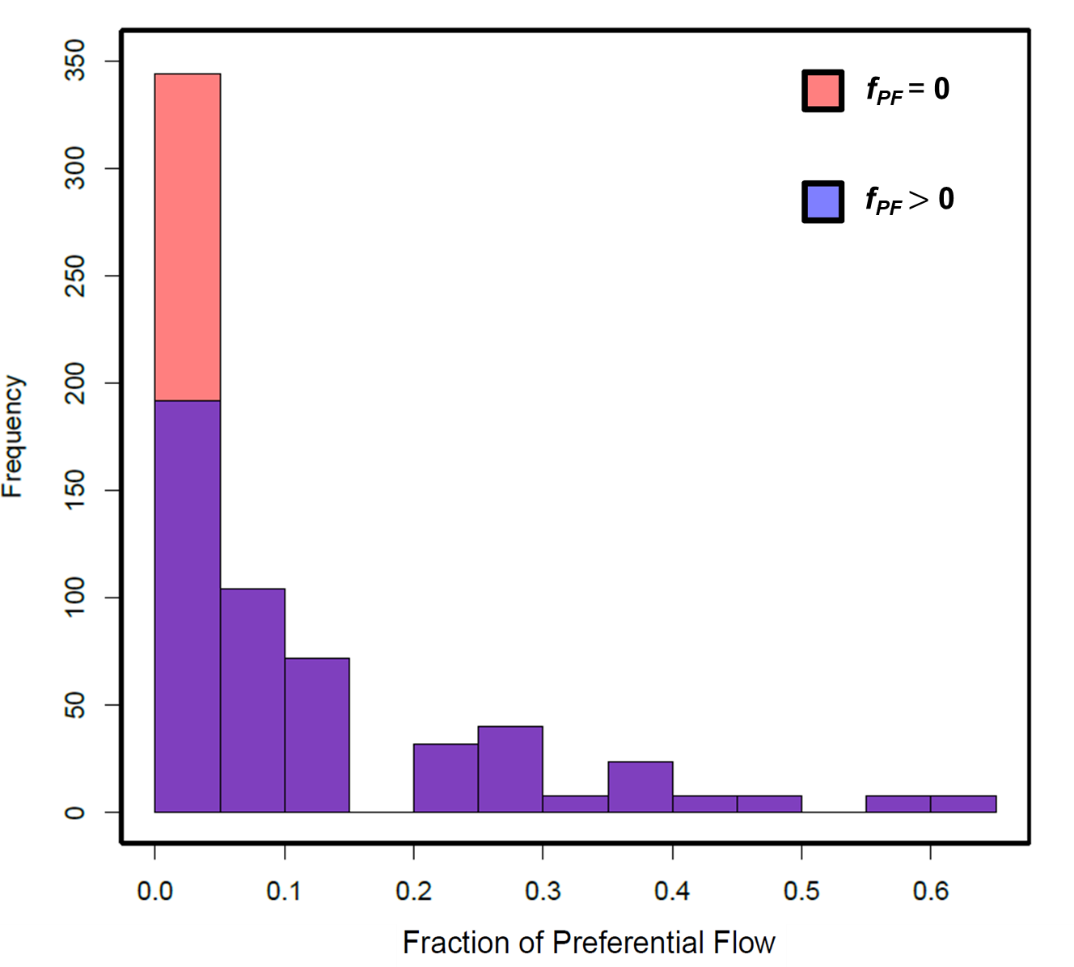
**

**Figure S1.** Experimental estimates of preferential flow using an isotope-mixing approach. Purple bars (*f_PF_* > 0) indicated samples in which the mixing model could detect preferential flow. The pink bar (*f_PF_* = 0) indicates samples in which the model could not distinguish event water from pre-event matrix water isotope signatures; these samples were removed from subsequent analyses. R v3.5.2 was used to plot this figure.^3^

**Distribution of Δ*C* versus *f_PF_***

Lysimeter data displayed a trend of increasing Δ*C* with preferential flow, though the relationship was noisy (**Figure S2** and **S3)**. For instance, high antibiotic concentrations were seen in samples without detectable preferential flow (e.g., ERY was measured with a Δ*C* value of 1.26 µg L^-1^) whereas some samples with high *f_PF_* had no detectable antibiotics (e.g., ERY, TYL, and TC). Positive Δ*C* detections were generally low in magnitude and clustered at low *f_PF_* values (**Figure S3**), indicating that bypass flow and leaching of antibiotics at high concentrations were relatively unlikely to occur.

**
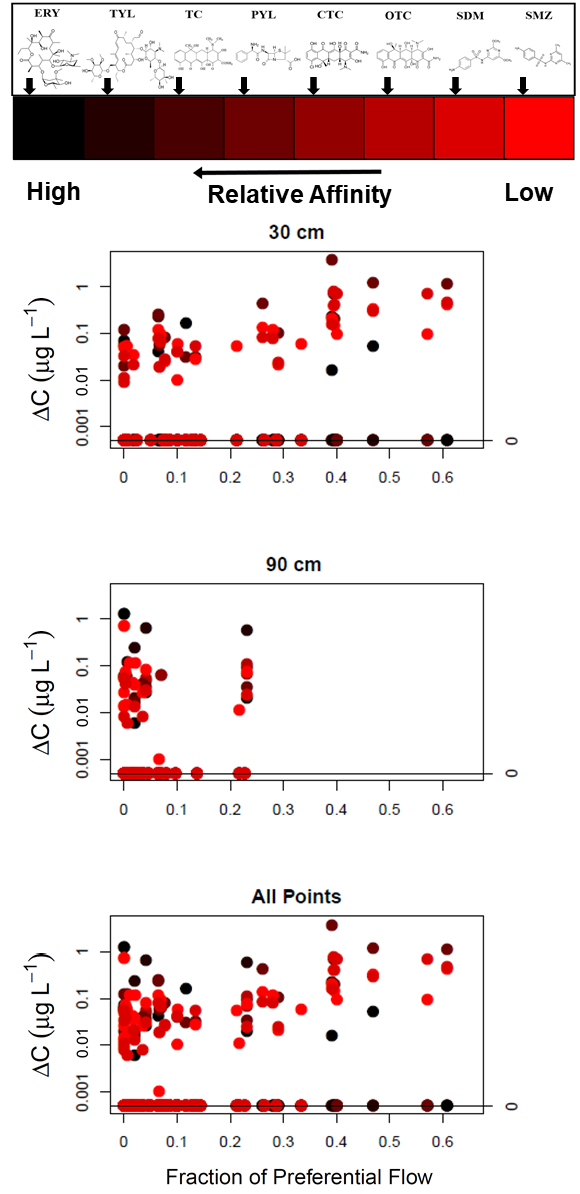
**

**Figure S2.** Changes in antibiotic concentrations in lysimeter samples before, during, and after the simulated rainfall versus fraction of preferential flow (non-detect samples included), with data divided between the (top) 30 cm sampling depth, (middle) 90 cm sampling depth, and (bottom) both depths combined. Colors indicate relative antibiotic affinity to soil as estimated from *K_d_* values: red indicates the lowest affinity and black indicates the highest affinity to soil. R v3.5.2 was used to plot this figure.^3^


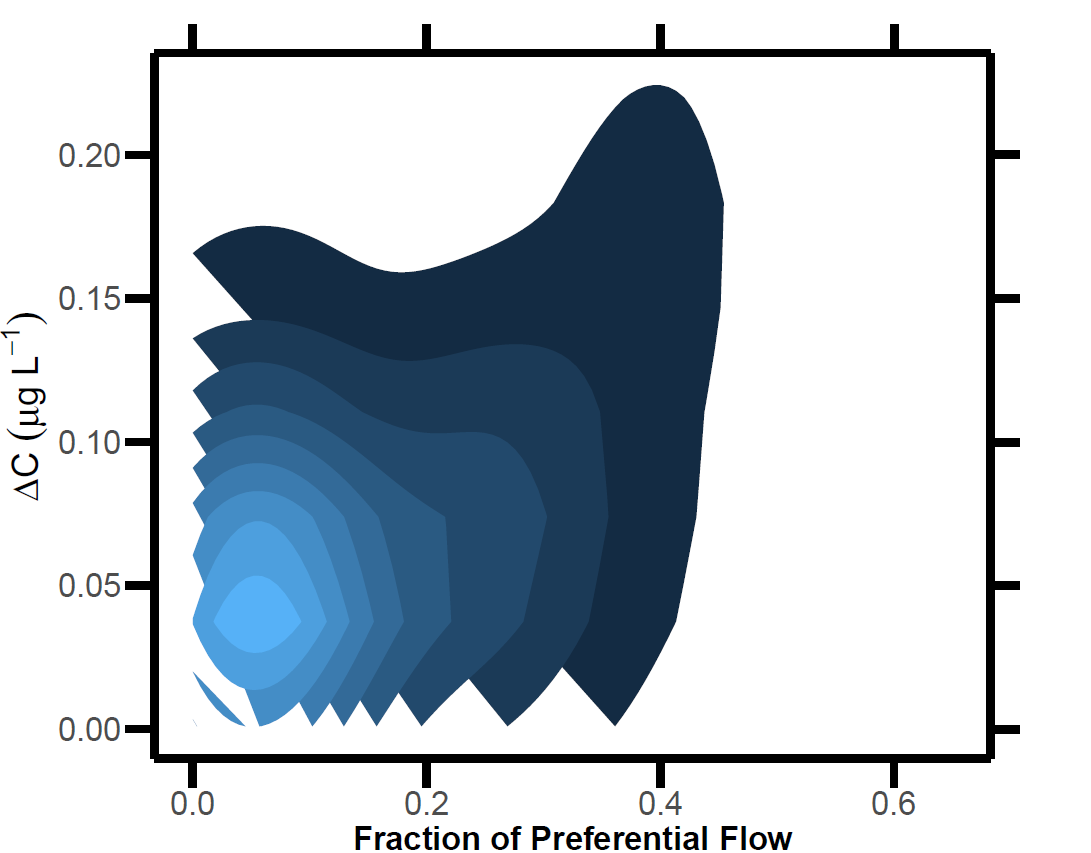


**Figure S3.** Bivariate kernel density plot depicting the distribution of results with positive change in antibiotic concentration (Δ*C*) and preferential flow values, where lightest areas are most probable and darkest areas are least probable. R v3.5.2 was used to plot this figure.^3^

**Distribution of Detectable (Δ*C* > 0) and Non-Detectable (Δ*C* = 0) Values**

**Figure S4** displays the distribution of antibiotic Δ*C* values used to produce **Figure 1a** in the main text (*f_PF_* > 0 and Δ*C* > 0). **Figure S5** shows that higher preferential flow estimates generally coincided with a lower occurrence of negative Δ*C* values. Also, the low-affinity sulfonamides, SDM and SMZ, were always detected with preferential flow exceeded 40%, whereas the other antibiotics all had multiple non-detects in that range.

**
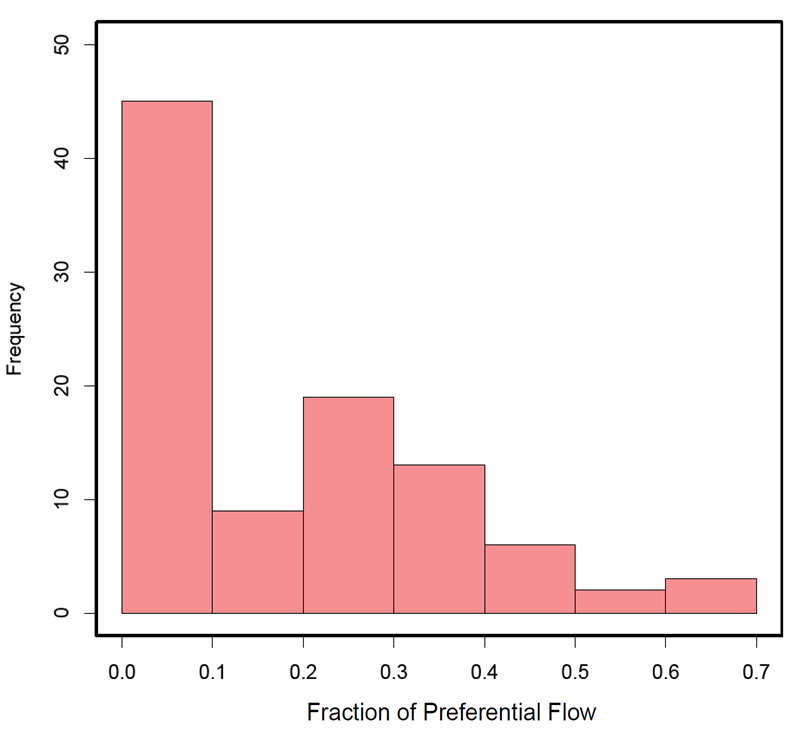
**

**Figure S4.** Histogram of fraction of preferential flow (*f_PF_*) values when Δ*C* > 0. R v3.5.2 was used to plot this figure.^3^


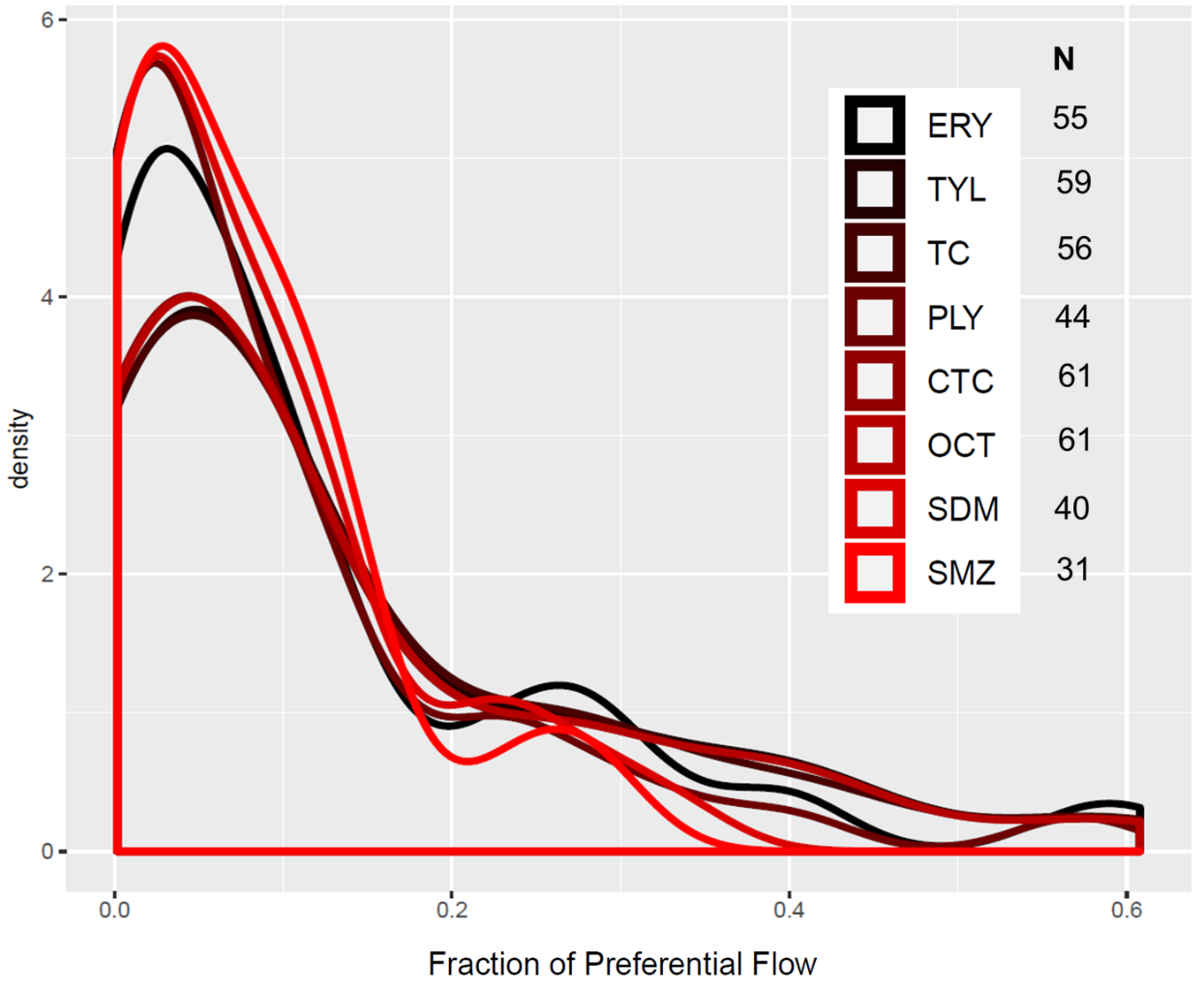


**Figure S5**. Smoothed density distributions of samples where preferential flow occurred (i.e., *f_PF_* > 0) yet antibiotics were not detected (i.e., Δ*C* = 0). N = the total number of points per compound that met the criteria Δ*C* = 0 and *f_PF_* > 0. Colors indicate their relative affinity to soil as ranked based on sorption (**Table S2)**: red indicates the lowest and black indicates the highest affinity to soil. R v3.5.2 was used to plot this figure.^3^

**Soil Colloidal Transport and Other Controlling Factors**

Here we recognize that the movement of numerous organic contaminants through a heterogeneous flow field (under non-equilibrium flow conditions) is highly complex, requiring *a priori* knowledge of chemical reactivity and empirical characterization of flow dynamics. Despite these complexities, our simplified relative affinity rankings provide unique insight into conditions where reactivity (i.e., compound-soil interactions) govern total transport. One possible explanation for this apparent muting of relative affinity control (e.g., in low range of preferential flow or moderate-affinity PLY showing highest Δ*C* in high *f_PF_* range) may be that moderate to high affinity antibiotics become susceptible to colloidal transport when sorbed to soil particles. This process, which is known to be an important transport mechanism for strongly sorbing antibiotics^4^ under steady state flow conditions, may intensify under preferential flow. Therefor the relative affinity of compounds to soil may be least influential in system that has both high preferential flow and low aggregate stability.^5^

**Assessment of Antibiotic Relative Affinity to Soil**

We performed a simple solute partitioning test to assess the relative affinity of our eight antibiotics to soil from the field site. One gram of dried and 2 mm sieved A_p_ horizon soil was mixed with 500 mL of deionized water to reach target concentrations of: 2.5 µg L^-1^ for SDM; 5 µg L^-1^ for SMZ, PLY, and TC; 50 µg L^-1^ for OTC, CTC, ERY, and TYL. These concentrations ranges and solution/soil ratios were chosen to be compatible with levels of antibiotic detection and the runoff solution/sediment ratio in samples taken previously from the same field.^6^ Each mixture was equilibrated for 1 h, vortexed, and shaken for 30 min. After shaking, the mixture was centrifuged at 5000 rpm for 5 min before a 1.5 mL aliquot of supernatant solution was mixed with 50 mg primary and secondary amine (PSA), vortexed, centrifuged, and sequentially filtered with a 0.45 µm PTFE syringe filter and a 0.2 µm PTFE syringe filter, before being injected into UPLC-MS/MS for analysis. Based on the magnitude of experimental determined or estimated *K_d_* values, a relative affinity ranking was assigned for each compound (**Table S5**)**.**

The two macrolides (ERY and TYL) were below the detection limits in the supernatant even after three additional tests at concentrations of 50, 250, and 500 µg L^-1^, so the experimental *K_d_* could not be calculated for those two compounds. This test suggested that the two compounds have much higher affinity to the soil matrix compared to other six compounds and were therefore assigned highest relative affinity rankings of 1 and 2 (**Table S5**).

**Sulfonamides in Control Plots**

Data from the control and experimental plots show that, in the presence of preferential flow, transport was similar for the sulfonamide veterinary antibiotics (SDM and SMZ), which had the lowest relative affinity for soil (**Figure S6**). Thus, after only 7 days of equilibration with the soil matrix, newly introduced antibiotics behaved similarly to residual compounds from manure applied in previous years. This result shows that these compounds with low matrix affinity are still susceptible to preferential flow despite long term persistence in the soil (i.e., > 6 months).

**Soil Sampling**

At the beginning of the experiment, intact cores (5 x 5 cm; *n* = 6 per horizon) and unconsolidated soil samples were taken from three soil horizons: Ap (5-10 cm), Bt1 (30-35 cm), and Bt2 (90-95 cm). Cores were used to determine soil bulk density [M L^-3^], porosity [L^3^ L^-3^], and saturated hydraulic conductivity (*K_s_*) [L T^-1^] while unconsolidated samples allowed for cation exchange capacity (CEC), pH, total organic carbon (TOC), and texture analysis (**Table S1**). Gravimetric soil water content was measured using samples collected from 0-5 cm depth, with sample collection occurring immediately prior to and after the rainfall simulations. Volumetric water content, *θ*, was calculated by multiplying gravimetric water content and bulk density of the Ap horizon (**Table S6**) and dividing by density of water (assumed to be 1 g cm^-3^). Core-derived *K_s_* was measured using the falling head method with a KSAT Benchtop Saturated Hydraulic Conductivity Instrument (UMS Inc., Munich, Germany). Unconsolidated soil samples were air dried, sieved to 2 mm, and analyzed for cation exchange capacity (CEC), pH, total organic carbon (TOC), and texture. CEC was measured via summation method,^7^ soil pH was measured in a 1:1 slurry (soil: CaCl_2_), TOC was quantified by dry combustion using a VarioMAX CNS elemental analyzer (Elementar, Hanau, Germany), and textural analysis was conducted via the pipet method.^8^ Soil physiochemical and hydraulic properties are shown in **Table S6**.


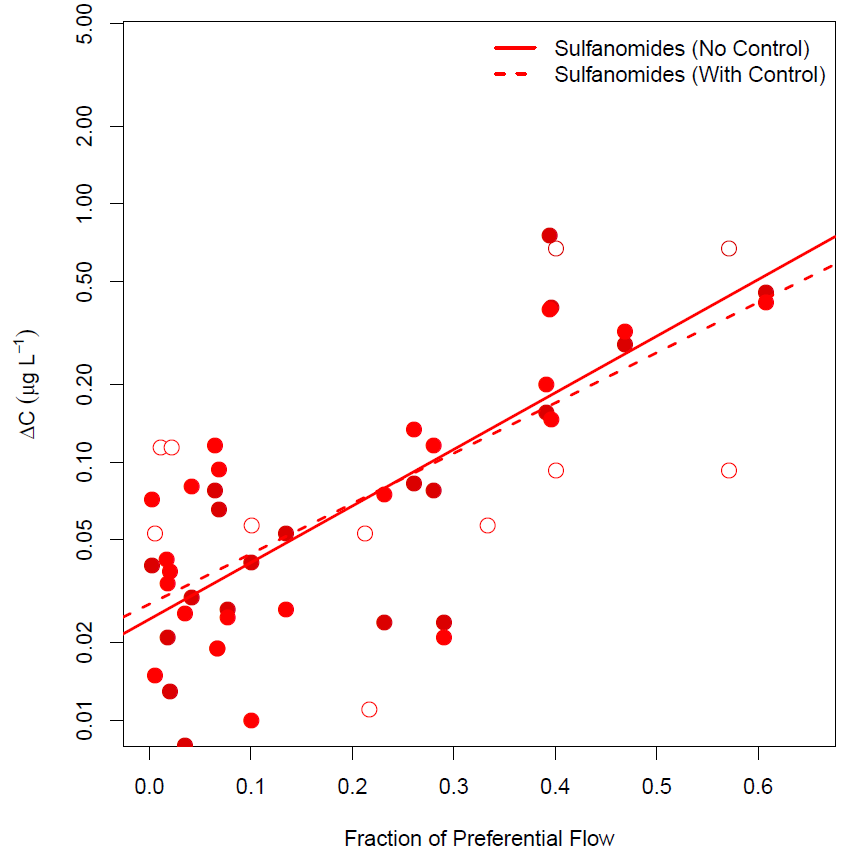


**Figure S6.** Changes in concentration (Δ*C*) during simulated rainfall of sulfonamides (SMZ and SDM) versus estimated fraction of preferential flow from lysimeter samples. Open circles represent samples from control plots, whereas filled circles represent plots where antibiotic-spiked manure was applied. Only samples with non-zero Δ*C* values were included. R v3.5.2 was used to plot this figure.^3^

**Comparison with Conventional Dual Permeability Simulations**

Here, we used the numerical platform HYDRUS-1D^9^ to simulate analogous conditions to our experimental design using the classical dual-domain model framework of Gerke and Van Genuchten.^10^ We applied solutes with relative affinity to the soil matrix that were either low (sorption coefficient, *K_d_*, of 64.6 cm^3^ g^-1^, to mimic SMZ) or high (*K_d_* of 1700 cm^3^ g^-1^, to mimic ERY), and set the sorption coefficient of the preferential flow domain to be 600x smaller for each solute (i.e., *K_d_*_,_*_F_* = 0.108 cm^3^ g^-1^ for the SMZ-like compound and 2.83 cm^3^ g^-1^ for the ERY-like solute). The system was allowed to equilibrate for 7 days, and we then applied simulated rainfall on Day 7. We created two physical model scenarios called “low preferential flow” and “high preferential flow” with realistic hydraulic parameters and different rainfall intensities to simulate a range of bypass flow reaching our 30 cm suction lysimeters. We further quantified the change in concentration of the preferential flow domain from 1 to 3 h following rainfall, and plotted these values against the fraction of simulated preferential flow (**Figure S7**). **Table S2**, **Table S3**, and **Table S4** provide detailed boundary conditions parameter descriptions, and parameter values for these simulations.

The simulation showed that the difference between solutes of high and low relative affinity decreased as the fraction of preferential flow increased. Even when varying the magnitude of important model parameters such as the solute mass transfer rate, we could not create a scenario in HYDRUS-1D in which differences in solute concentration between low and high relative affinity compounds increased as preferential flow increased (i.e., the behavior observed in our field study). Therefore, this simulation further emphasizes that our study results were unexpected and do not easily or fully conform to established models or theory.

**
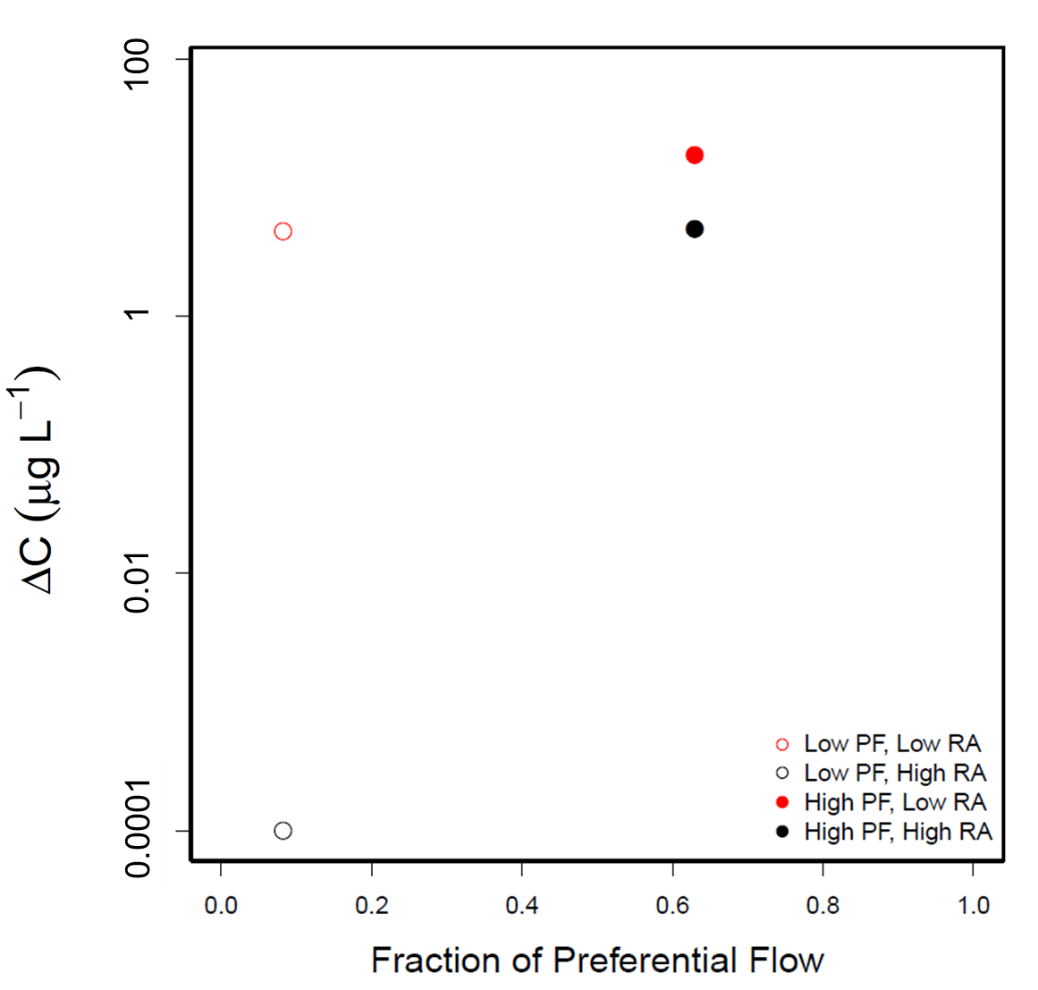
**

**Figure S7.** Change in solute concentration versus fraction of preferential flow following rainfall simulated by dual-permeability module of HYDRUS-1D. Data come from 30 cm depth and represent 1 to 1.5 h after initiation of rainfall. We created two physical model scenarios called “low preferential flow” (Low PF) and “high preferential flow” (High PF) using realistic hydraulic parameters. Two solutes were modeled, including one with low relative affinity to the soil matrix (Low RA) and one with high relative affinity to the soil matrix (High RA).

**Table S2.** Boundary conditions HYDRUS simulations.

|  | **Low PF,**  **Low RA** | **Low PF,**  **High RA** | **High PF,**  **Low RA** | **High PF,**  **High RA** |
| --- | --- | --- | --- | --- |
| Rain Intensity at 169-170 h (cm h^-1^) | 4.3 | 4.3 | 10 | 10 |
| ET (cm h^-1^) | 0 | 0 | 0 | 0 |
| Lower Boundary | Free Drainage | Free Drainage | Free Drainage | Free Drainage |

**Table S3.** Parameters descriptions for HYDRUS simulations.

| θ_r_ (cm^3^ cm^-3^) | residual water content of matrix |
| --- | --- |
| θ_s_ (cm^3^ cm^-3^) | saturated water content of matrix |
| α (cm^-1^) | van Genuchten parameter for the matrix |
| n (-) | van Genuchten parameter for the matrix |
| K_s_ (cm h^-1^) | Saturated hydraulic conductivity of the matrix |
| τ (-) | tortuosity factor in the matrix conductivity function |
| θ_r,F_ (cm^3^ cm^-3^) | residual water content of fractures |
| θ_s,F_ (cm^3^ cm^-3^) | saturated water content of fractures |
| α_F_ (cm^-1^) | van Genuchten parameter of fractures |
| n_F_ | van Genuchten parameter of fractures |
| K_s,F_ (cm h^-1^) | Saturated hydraulic conductivity of the fractures |
| τ_F_ (-) | tortuosity factor in the fracture conductivity function |
| ω (-) | ratio of the volume of fractures versus total porosity |
| β (-) | geometric shape factor |
| γ (-) | scaling factor |
| a (cm) | effective diffusion path length |
| K_s,a_ (cm h^-1^) | effective hydraulic conductivity at the fracture-matrix interface |
| ρ_b_ (g cm^-3^) | soil bulk density |
| λ_L_ (cm) | longitudinal dispersivity of the matrix |
| Fr.M (-) | fraction of adsorption sites with instantaneous sorption in the matrix |
| θ_im_ (cm^3^ cm^-3^) | immobile water content of the matrix |
| D_w_ (cm^2^ h^-1^) | molecular diffusion coefficient in free water |
| Г_s_ (cm^2^ h^-1^) | solute mass transfer coefficient for transfer between matrix and fracture domains |
| K_d_ (L kg^-1^) | equilibrium sorption coefficient of the matrix |
| ν (L kg^-1^) | Langmuir coefficient |
| Beta | Freundlich exponent |
| AlphaM (h^-1^) | first order rate coefficient for one or two site in the matrix, equilibrium or nonequilibrium adsorption |
| SinkWater1 (h^-1^) | First-order rate constant for dissolved phase |
| Sinksolid1 (h^-1^) | First-order rate constant for solid phase |
| λ_L,F_ (cm) | longitudinal dispersivity of the fractures |
| SinkWater1' (h^-1^) | First-order rate constant for dissolved phase for chain reaction |
| SinkSolid1' (h^-1^) | First-order rate constant for solid phase for chain reaction |
| Fr.F | fraction of adsorption sites with instantaneous sorption in the fractures |
| SinkWater0 (h^-1^) | First-order rate constant for dissolved phase for alternative chain reaction |
| SinkSoil0 (h^-1^) | First-order rate constant for solid phase for alternative chain reaction |
| K_d,F_ (L kg^-1^) | equilibrium sorption coefficient of the fractures |
| AlphaF | first order rate coefficient for one or two site in the fractures equilibrium or nonequilibrium adsorption |

**Table S4.** Parameters values for HYDRUS simulations.

|  | **Low PF, Low RA** | **Low PF, High RA** | **High PF, Low RA** | **High PF, High RA** |
| --- | --- | --- | --- | --- |
|  | *Hydraulic Properties* | | | |
| θ_r_ (cm^3^ cm^-3^) | 0 | 0 | 0 | 0 |
| θ_s_ (cm^3^ cm^-3^) | 0.4 | 0.4 | 0.4 | 0.4 |
| α (cm^-1^) | 0.05 | 0.05 | 0.05 | 0.05 |
| n (-) | 1.5 | 1.5 | 1.5 | 1.5 |
| K_s_ (cm h^-1^) | 3.6 | 3.6 | 3.6 | 3.6 |
| τ (-) | 0.5 | 0.5 | 0.5 | 0.5 |
| θ_r,F_ (cm^3^ cm^-3^) | 0 | 0 | 0 | 0 |
| θ_s,F_ (cm^3^ cm^-3^) | 1 | 1 | 1 | 1 |
| α_F_ (cm^-1^) | 6 | 6 | 4 | 4 |
| n_F_ | 2 | 2 | 1.8 | 1.8 |
| K_s,F_ (cm h^-1^) | 12 | 12 | 48 | 48 |
| τ_F_ (-) | 1 | 1 | 1 | 1 |
| ω (-) | 0.1 | 0.1 | 0.25 | 0.25 |
| β (-) | 1 | 1 | 1 | 1 |
| γ (-) | 1 | 1 | 1 | 1 |
| a (cm) | 0.1 | 0.1 | 0.1 | 0.1 |
| K_s,a_ (cm h^-1^) | 0 | 0 | 0 | 0 |
|  | *Transport Properties* | | | |
| ρ_b_ (g cm^-3^) | 1.5 | 1.5 | 1.5 | 1.5 |
| λ_L_ (cm) | 2 | 2 | 2 | 2 |
| Fr.M (-) | 1 | 1 | 1 | 1 |
| θ_im_ (cm^3^ cm^-3^) | 0 | 0 | 0 | 0 |
| D_w_ (cm^2^ h^-1^) | 2x10^-0.005^ | 2x10^-0.005^ | 2x10^-0.005^ | 2x10^-0.005^ |
| Г_s_ (cm^3^ h^-1^) | 0.002 | 0.002 | 0.002 | 0.002 |
|  | *Reaction Properties* | | | |
| K_d_ (cm^3^ g^-1^) | 64.6 | 1700 | 64.6 | 1700 |
| ν (cm^3^ g^-1^) | 0 | 0 | 0 | 0 |
| Beta | 1 | 1 | 1 | 1 |
| AlphaM (h^-1^) | 0 | 0 | 0 | 0 |
| SinkWater1 (h^-1^) | 0 | 0 | 0 | 0 |
| Sinksolid1 (h^-1^) | 0 | 0 | 0 | 0 |
| λ_L,F_ (cm) | 1 | 1 | 1 | 1 |
| SinkWater1' (h^-1^) | 0 | 0 | 0 | 0 |
| SinkSolid1' (h^-1^) | 0 | 0 | 0 | 0 |
| Fr.F | 1 | 1 | 1 | 1 |
| SinkWater0 (h^-1^) | 0 | 0 | 0 | 0 |
| SinkSoil0 (h^-1^) | 0 | 0 | 0 | 0 |
| K_d,F_ (cm^3^ g^-1^) | 0.108 | 2.83 | 0.108 | 2.83 |
| AlphaF | 0 | 0 | 0 | 0 |

**Analytical Approach**

Each collected leachate sample was first cleaned up by mixing 1.8 mL leachate with 60 mg primary and secondary amine (PSA), vortexing for 2 min, and centrifuging at 5000 rpm for 5 min. The supernatant was then filtered first through a 0.45 µm PTFE syringe filter and second through a 0.2 µm PTFE syringe filter immediately before being analyzed using an online solid phase extraction (SPE) system coupled with a Ultra-Performance Liquid Chromatography/tandem mass spectrometry (UPLC/MS/MS) (Agilent 1290 Infinity LC system with Flexible Cube, using an Agilent 6490 Triple Quadrupole system (Agilent, Santa Clara, CA). Six hundred µL of a filtered leachate was loaded onto the online SPE installed with Agilent PLRP-S cartridges (15-20 µm, 4.6 × 12.5mm, Agilent). Cartridges were eluted with 100% water at 0.1 min, 100% acetonitrile at 4.6 min, and 100% water again at 13 min. all with a constant flow rate of 1 mL min^-1^. Separation of target analytes was conducted on a Zorbax SB-C18 analytical column (3.0 × 100 mm, 3.5-µm particle size, Agilent) at 40^o^C with a mobile phase flow rate of 0.4 mL min^-1^. A Zorbax SB-C18 guard column (2.1 × 50 mm, 1.8-µm particle size, Agilent) was installed before the analytical column to remove matrix interference. The mobile phase included (A) 0.1% formic acid in water and (B) 90% acetonitrile with the following A:B gradients of 80:20, 80:20, 80:20, 39:61, 0:100, 0:100, and 80:20, measured at respective times of 0, 0.1, 4.6, 11.1, 12.6, 14.1, and 14.6 min.

**Table S5.** Veterinary antibiotics with experimentally determined sorption coefficient (*K_d_*), relative affinity ranking with 1 being the highest and 8 being the lowest, and EPI (estimation program interface) predicted half-lives.

| Veterinary Antibiotic (VA) | *K_d_* (L kg^-1^) | Relative Affinity | EPI Predicted Half Life (d) |
| --- | --- | --- | --- |
| Eryrthomycin (ERY) | *NA* | 1 | 360 |
| Tylosin (TYL) | *NA* | 2 | 360 |
| Tetracycline (TC) | 1140 | 3 | 120 |
| Pirlimycin (PLY) | 997 | 4 | 75 |
| Chlortetracycline (CTC) | 783 | 5 | 360 |
| Oxytetracycline (OTC) | 368 | 6 | 120 |
| Sulfadimethoxam (SDM) | 80.3 | 7 | 75 |
| Sulfamethazine (SMZ) | 64.6 | 8 | 75 |

**Table S6.**  Summary of soil physiochemical and hydraulic properties. Note that cation exchange capacity (CEC), porosity, bulk density, saturate hydraulic conductivity (K_s_), pH and total organic carbon content (TOC) are expressed as mean ± standard deviation.

| Horizon | Depth (cm) | Texture | Sand (%) | Silt (%) | Clay (%) | CEC  (cmol kg^-1^) | Porosity (%) | Bulk density  (g cm^-3^) | K_s_ (cm d^-1^) | pH | TOC (%) |
| --- | --- | --- | --- | --- | --- | --- | --- | --- | --- | --- | --- |
| Ap | 0-23 | Loam | 44.5 | 41.6 | 25.8 | 16 ± 4.6 | 47± 0.01 | 1.36 ± 0.05 | 416 ± 486 | 6.0 ± 0.26 | 2.0 ± 0.45 |
| Bt1 | 23-33 | Sandy clay loam | 45.1 | 46.9 | 29.1 | 4.4 ± 1.7 | 37 ± 0.04 | 1.75 ± 0.13 | 43.8 ± 50.8 | 5.6 ± 0.27 | 0.25 ± 0.062 |
| Bt2 | 33-97 | Silty clay | 10.2 | 11.4 | 45.0 | 8.1 ± 0.30 | 54 ± 0.07 | 1.4 ± 0.04 | 44 ± 45.3 | 4.9 ± 0.07 | 0.40 ± 0.031 |

The tandem mass spectrometry system was set up using an electrospray ionization positive ion mode at nitrogen gas temperature of 250^o^C, nitrogen gas flow of 14 L min^-1^, nebulizer gas pressure of 310 kPa, and capillary voltage of 3500 V. The mass to charge ratios (m/z) for the parent ion and qualifier and quantifier daughter ions are listed in **Table S7**. Due to significant matrix interference, concentrations of all target analytes in the leachate samples were quantified against matrix-matched standards.^11^ The method detection limits and recoveries of the target analytes are listed in **Table S8**.

All liquid samples (i.e., pore water from lysimeters and column leachate) were also analyzed for ^18^O and ^2^H via cavity ring down spectroscopy (Model L1102-i, Picarro, Santa Clara, CA). All samples were expressed in per mil (‰) delta notation relative to Vienna Mean Standard Mean Ocean Water (VSMOW) via

δ^18^O or δ^2^H = $\left( \frac{R_{sample}}{R_{VSMOW}}-1 \right)\times1000$ (1)

where *R_sample_* and *R_VSMOW_* are the respective ratios of heavy to light species in the sample and standard VSMOW water.

We used a modified post-processing method^12^ to apply drift and memory corrections to raw data, yielding a precision of 0.1‰ for ^18^O and 0.2‰ for ^2^H. Analysis of the input water samples showed δ^2^H = 1.08 x 10^2^ ‰ for the spiked rainwater and δ^2^H = -41 ‰ for the well water.

**Table S7.** Mass to charge (m/z) of the parent ion and daughter ions for target analytes

| Antibiotic | Parent Ion (m/z) | Qualifier Daughter Ion (m/z) | Quantifier Daughter Ion (m/z) |
| --- | --- | --- | --- |
| Tylosin (TYL) | 916.0 | 772.0 | 174.0 |
| Pirlimycin (PYL) | 411.0 | 363.0 | 112.0 |
| Tetracycline (TC) | 445.2 | 154.0 | 410.0 |
| Erythromycin (ERY) | 734.6 | 576.5 | 158.2 |
| Oxytetracycline (OTC) | 461.6 | 443.0 | 426.0 |
| Sulfadimethoxine (SDM) | 311.2 | 92.1 | 156.1 |
| Sulfamethazine (SMZ) | 279.0 | 156.0 | 186.0 |
| Chlorotetracycline (CTC) | 479.1 | 153.9 | 443.9 |

**Table S8.** Method detection limit (MDL) and recovery of target compounds in their matrix

| Antibiotic | MDL  (ng L^-1^) | Recovery (%)  (*n* =10) |
| --- | --- | --- |
| Tylosin (TYL) | 249 | 102 ± 14 |
| Pirlimycin (PYL) | 12.5 | 100 ± 8 |
| Tetracycline (TC) | 6.39 | 108 ± 14 |
| Erythromycin (ERY) | 6.25 | 90 ± 12 |
| Oxytetracycline (OTC) | 124 | 111 ± 7 |
| Sulfadimethoxine (SDM) | 25.7 | 102 ± 9 |
| Sulfamethazine (SMZ) | 6.35 | 96 ± 13 |
| Chlorotetracycline (CTC) | 63.2 | 91 ± 7 |

**Field Plot Design and Lysimeter Placement**

**Figure S8** shows the design of field plots that received antibiotic-spiked manure via surface application or subsurface injection, along with their respective lysimeter placement schemes. Control plot design and lysimeter placement were identical to surface application plots.


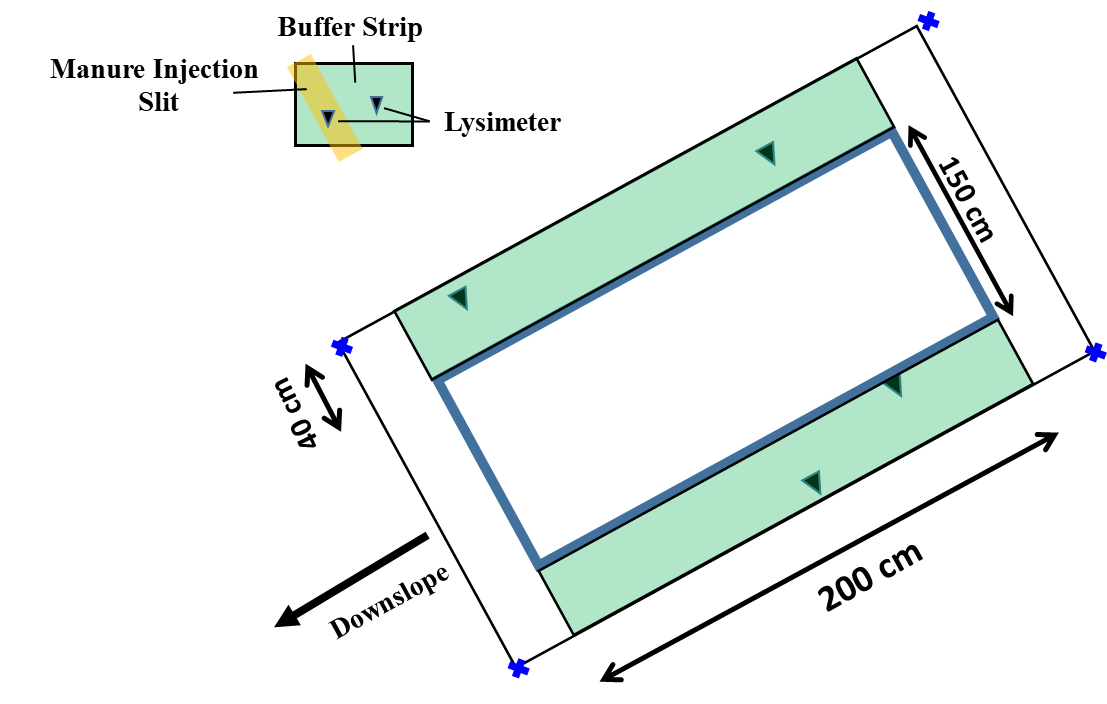

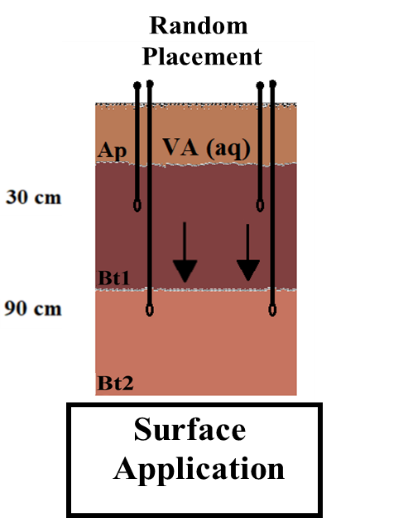

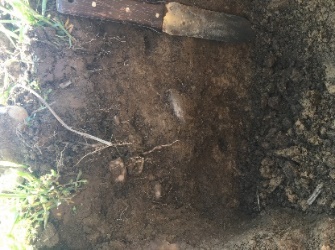

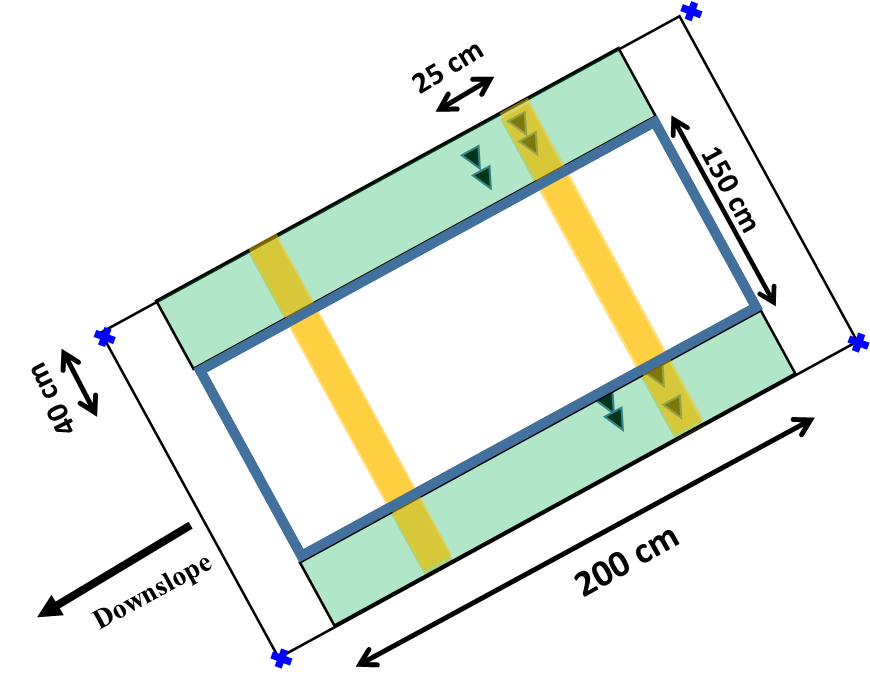

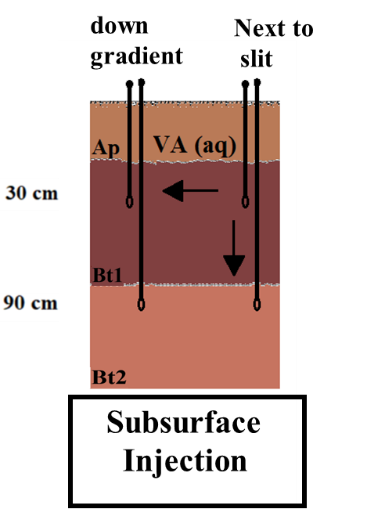


x 2

**Figure S8.** Schematic showing the basic design for subsurface injection (**top**) and surface application (**bottom**) plots along with their respective lysimeter installation schemes. The center photograph shows soil structure development in the A and B horizons. Note: Control plots were designed and sampled in an identical manner to the Surface Application plots.

**Background Detection of Antibiotics**

**Figure S9** shows background concentrations antibiotics in lysimeters taken 1 h prior to rainfall simulations.

**
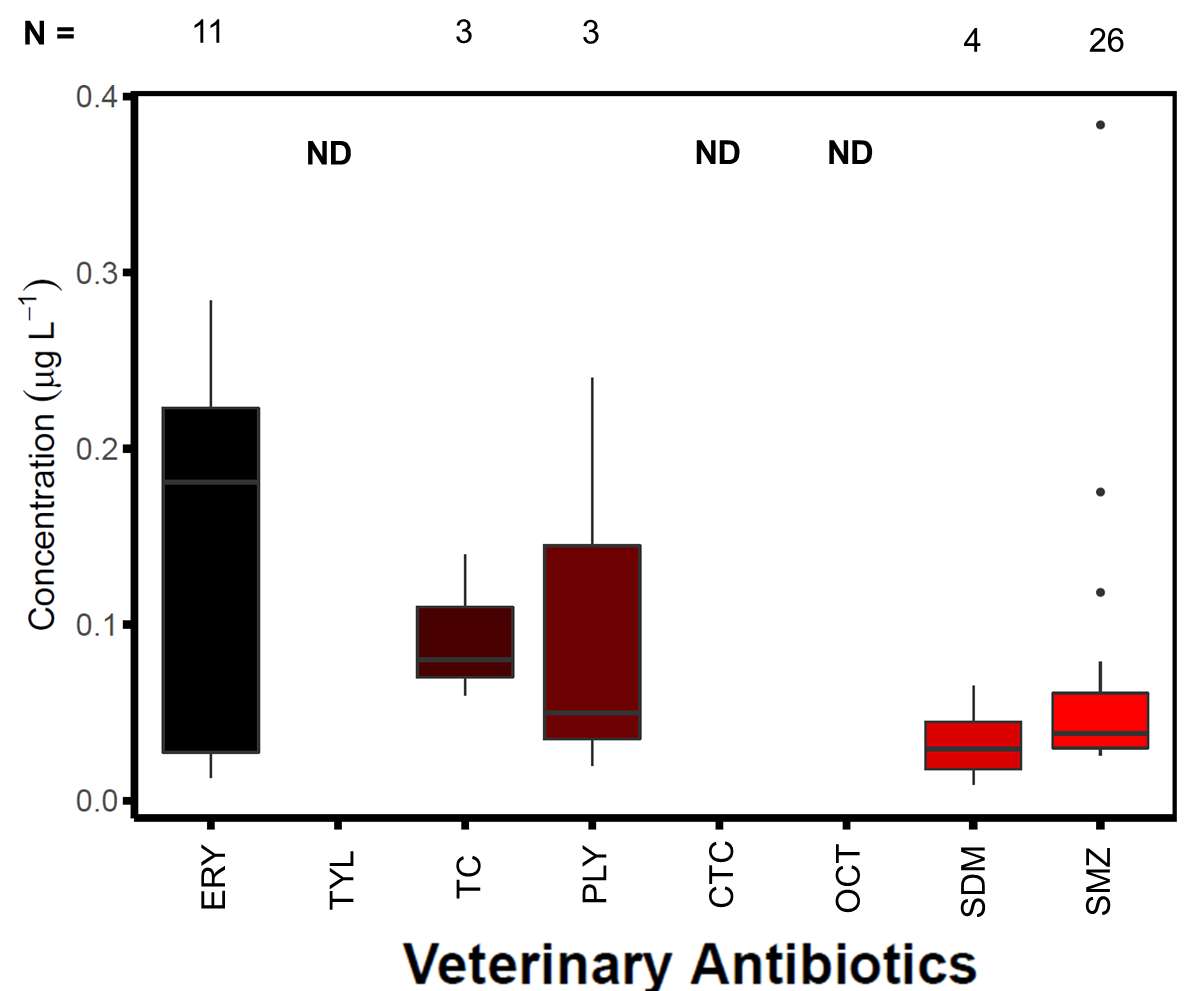
**

**Figure S9**. Background concentrations of antibiotics in lysimeter samples taken 1 h before rainfall simulations. Colors indicate their relative affinity to soil as ranked based on the sorption study (*K_d_* values listed in **Table S3**). **Above**) number of samples yielding detectable antibiotics are listed, whereas **ND** denotes non-detection. Median values are 0.18, 0.09, 0.1, 0.03, and 0.06 µg L^-1^ for ERY, TC, PLY, SDM, and SMZ, respectively. R v3.5.2 was used to plot this figure.^3^

**Lysimeter Sampling Details**

**Figure S10** shows the lysimeter sampling scheme during rainfall simulations.


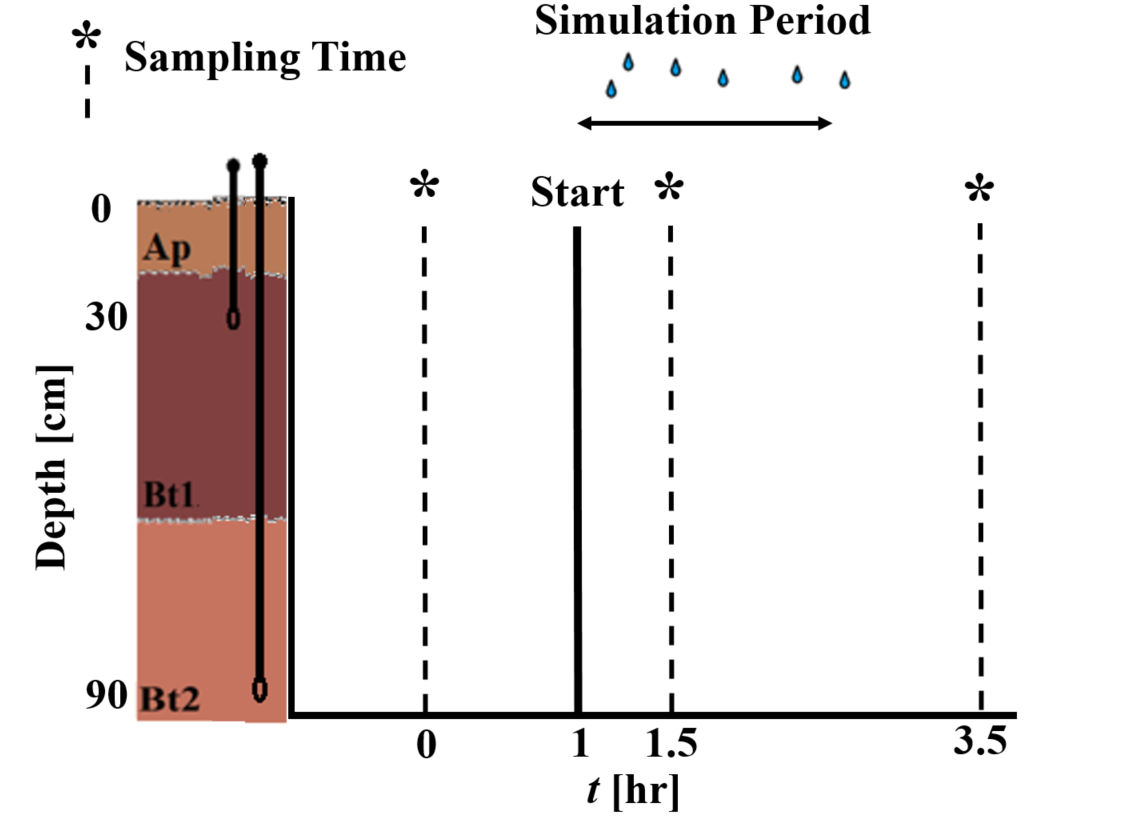


**Figure S10.** Lysimeter sampling regiment for field rainfall simulations, including two sampling depths (30 and 90 cm) and three sampling times (1 hour before, 0.5 hours into, and 1 hour after rainfall simulations).

**Manure Treatment Comparisons and Sampling Time**

Linear models fit to subsurface injection, surface application and control treatments (excluding Δ*C* = 0 and *f_PF_* = 0 values) had slopes of 1.85 (subsurface injection), 2.78 (surface application), and 1.06 (control). Adjusted r^2^ values were 0.27 for subsurface injection, 0.48 for surface application, and 0.11 for control plots. We found no significant difference between the slope of lines fitted to Δ*C* data across the range of *f_PF_* for all treatments, and no significant influence of lysimeter depth on this relationship (ANCOVA, *p* > 0.05; **Figure S11**). Therefore, because antibiotic transport was similar regardless of treatment type or depth, we compiled all data together for subsequent analyses. Additionally, because 1) high variation in *K*_s_ across our field (**Table S6**) suggested that intrinsic flow heterogeneity would mask any influence of time on Δ*C*, and 2) there appears to be no consistent effect of sampling time on the relationship between Δ*C* and *f_PF_* (**Figure S12**), we considered the effect of sampling time to be negligible.


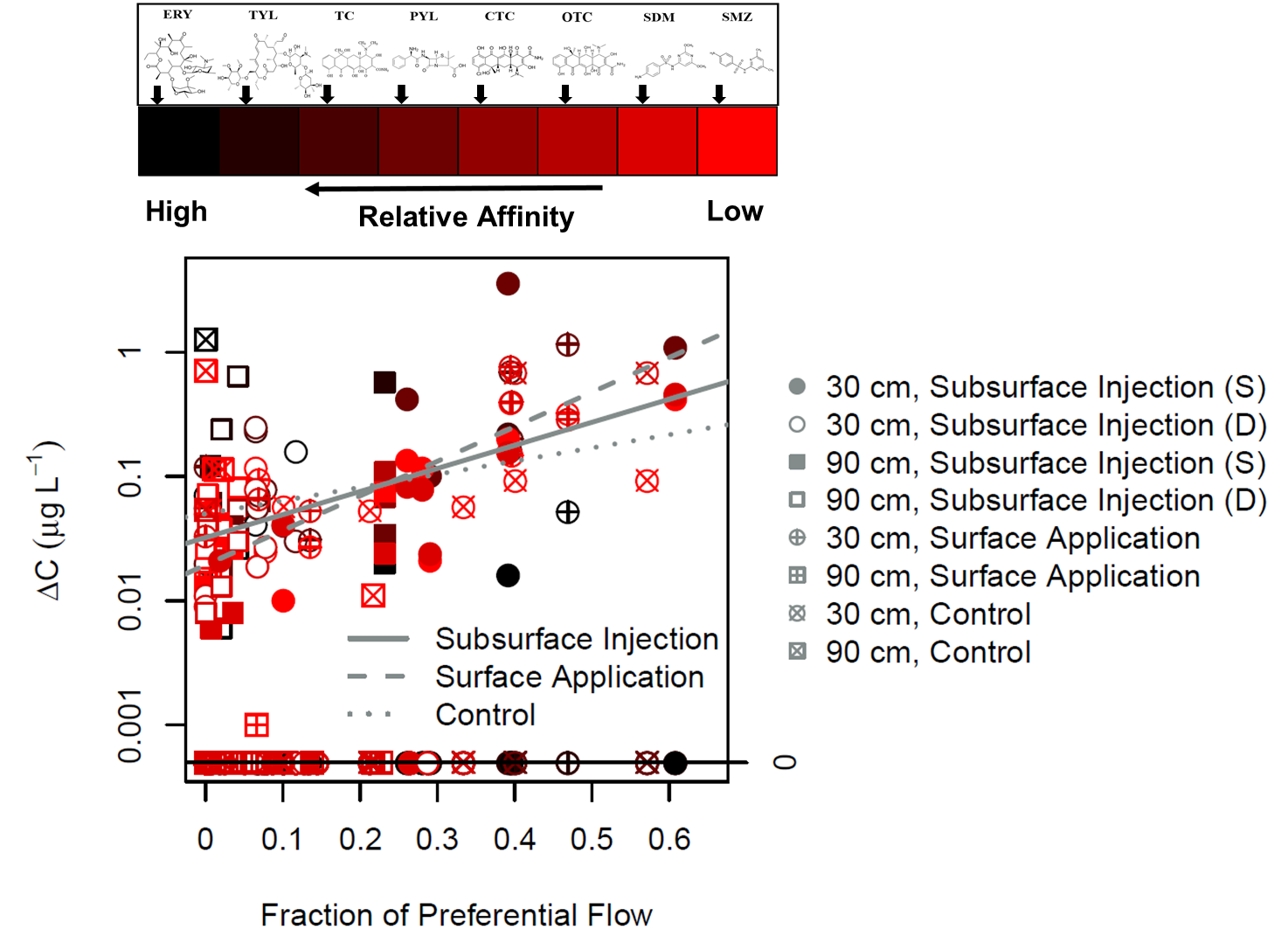


**Figure S11.**  Change in veterinary antibiotic concentration (Δ*C*) versus fraction of preferential flow (*f_pf_*) estimated in lysimeter samples (Δ*C* = 0 and *f_PF_* = 0 included). Colors indicate the relative affinity to soil as ranked based on the sorption study (*K_d_* values listed in **Table S3)**: red indicates the lowest affinity and black indicates the highest affinity to soil. “S” refers to samples taken from lysimeters within injection slits, while “D” represents samples collected from lysimeters down-gradient of the injection slits. Linear models were fit to Subsurface Injection, Surface Application and Control data points (Δ*C* = 0 and *f_PF_* = 0 excluded). Note that the overall and treatment specific (Δ*C*) vs *f_PF_* relationships were statistically significant (*p* < 0.001). R v3.5.2 was used to plot this figure.^3^

**
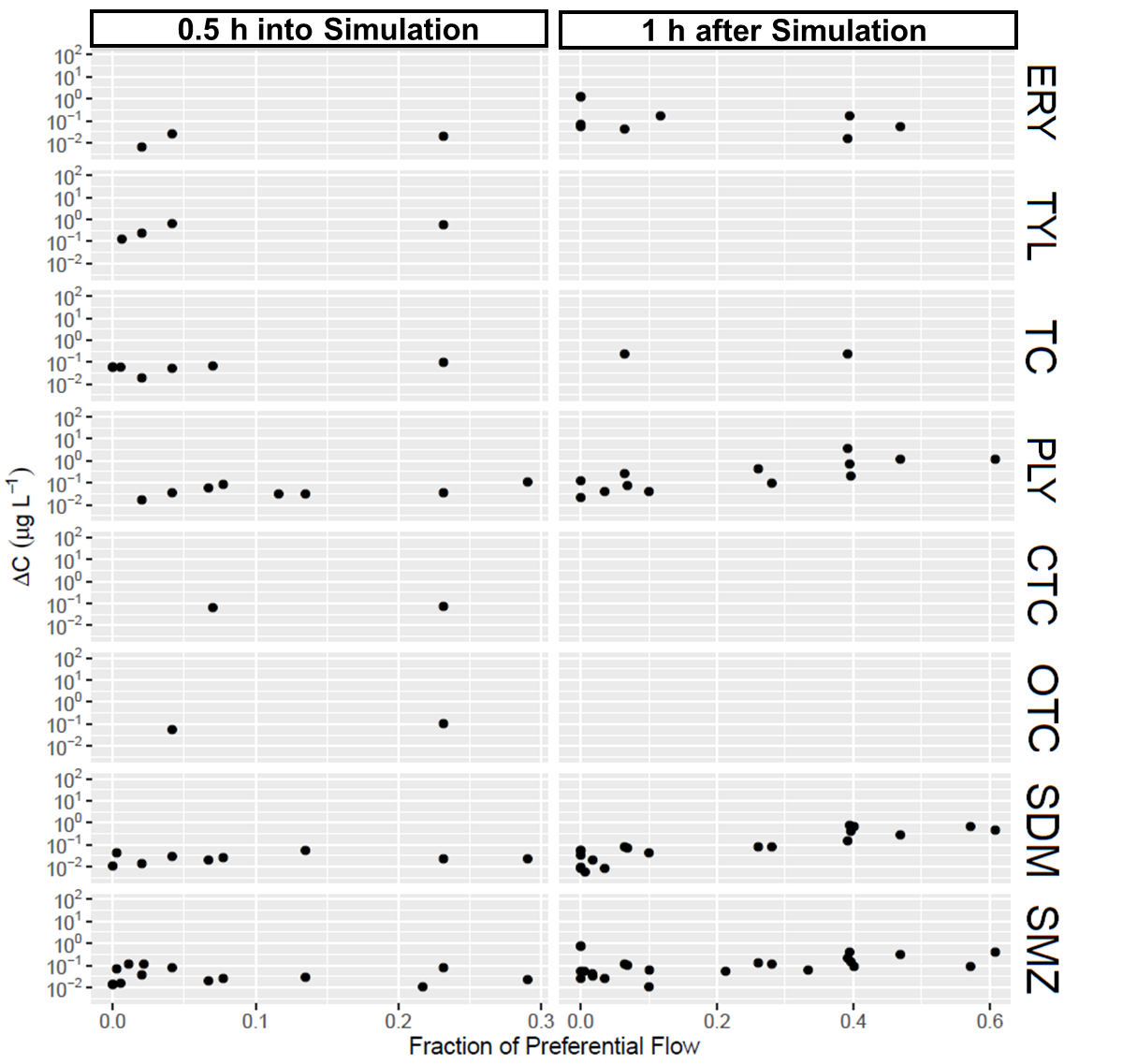
**

**Figure S12.**  Change in veterinary antibiotic concentration (Δ*C*) versus fraction of preferential flow (*f_pf_*) estimated in lysimeter samples (Δ*C* > 0 and *f_PF_* > 0), separated by sampling time and antibiotic.

**Water Samples Taken**

A total of 144 (48 lysimeters x 3 sampling points) lysimeter water samples were taken during the course of the experiment. Of these, a maximum of 96 observations were used to further investigate transport dynamics (2 effective measurement periods x 48 lysimeters) by producing ΔC and *f_PF_* values. Since we analyzed each water sample for 8 separate compounds and subsequent analyses consider all 8 antibiotics, the effective 96 samples can be multiplied times 8 to yield a total of 768 measurements considered for ΔC and *f_PF_*. Along with this document we provide the full list of samples taken for this experiment containing treatment information, lysimeter position, and sample timing along with associated antibiotic and stable isotope delta values. See the spreadsheet entitled “Radolinski_et_al_dC_PF_spectrum_data_v2.csv.” Note that that the experimental observation period extended through three sampling periods (0 < “Obs.Number” < 4). Columns “C_rain” and “C_matrix” correspond to the δ^2^H (‰) values of simulated rainfall and soil matrix porewater respectively used to calculate *f_PF_*.

**References**

1 Allen, S. T., Kirchner, J. W., Braun, S., Siegwolf, R. T. & Goldsmith, G. R. Seasonal origins of soil water used by trees. *Hydrology and Earth System Sciences* **23**, 1199-1210 (2019).

2 Zhang, Z., Si, B., Li, H. & Li, M. Quantify Piston and Preferential Water Flow in Deep Soil Using Cl− and Soil Water Profiles in Deforested Apple Orchards on the Loess Plateau, China. *Water* **11**, 2183 (2019).

3 Team, R. C. Vol. 3.5.2 (R Foundation for Statistical Computing

Vienna, Austria, 2020).

4 Xing, Y., Chen, X., Wagner, R. E., Zhuang, J. & Chen, X. Coupled effect of colloids and surface chemical heterogeneity on the transport of antibiotics in porous media. *Science of The Total Environment*, 136644 (2020).

5 Li, F. *et al.* Enhanced soil aggregate stability limits colloidal phosphorus loss potentials in agricultural systems. *Environmental Sciences Europe* **32**, 17 (2020).

6 Le, H. T., Maguire, R. O. & Xia, K. J. J. o. E. Q. Method of dairy manure application and time before rainfall affect antibiotics in surface runoff. (2018).

7 Hajek, B., Adams, F. & Cope, J. Rapid Determination of Exchangeable Bases, Acidity, and Base Saturation for Soil Characterization 1. *Soil Science Society of America Journal* **36**, 436-438 (1972).

8 Day, P. R. Particle fractionation and particle-size analysis. *Methods of soil analysis. Part 1. Physical and mineralogical properties, including statistics of measurement and sampling* **9**, 545-567 (1965).

9 Simunek, J., Sejna, M., Saito, H. & Th. Van Genuchten, M. *The HYDRUS-1D Software Package for Simulating the One-Dimensional Movement of Water, Heat, and Multiple Solutes in Variably-Saturated Media* (UJniversity of California Riverside, 2009).

10 Gerke, H. H. & Van Genuchten, M. T. A dual‐porosity model for simulating the preferential movement of water and solutes in structured porous media. *Water resources research* **29**, 305-319 (1993).

11 Commission, E. Commission Decision of 12 August 2002 implementing Council Directive 96/23/EC concerning the performance of analytical methods and the interpretation of results. *Off. J. Eur. Commun.* **221**, 8-36 (2002).

12 van Geldern, R., Barth, J. A. J. L. & Methods, O. Optimization of instrument setup and post‐run corrections for oxygen and hydrogen stable isotope measurements of water by isotope ratio infrared spectroscopy (IRIS). **10**, 1024-1036 (2012).
